# Venturicidin A, A Membrane-active Natural Product Inhibitor of ATP synthase Potentiates Aminoglycoside Antibiotics

**DOI:** 10.1038/s41598-020-64756-0

**Published:** 2020-05-18

**Authors:** Venkateswarlu Yarlagadda, Ricardo Medina, Gerard D. Wright

**Affiliations:** 1 David Braley Centre for Antibiotic Discovery, M.G. DeGroote Institute for Infectious Disease Research, Department of Biochemistry and Biomedical Sciences, DeGroote School of Medicine, McMaster University, 1280 Main Street West, Hamilton Ontario, L8S 4K1 Canada; 2 Department of Microbiology, Central University of Las Villas, Santa Clara, Villa Clara Cuba

**Keywords:** Drug screening, Antimicrobials, Antibiotics, Antimicrobial resistance, Mechanism of action, Natural products, Screening, Biochemistry, Chemical biology, Drug discovery, Microbiology, Chemistry, Chemical biology, Mechanism of action, Natural products, Screening

## Abstract

Despite the remarkable advances due to the discovery and development of antimicrobials agents, infectious diseases remain the second leading cause of death worldwide. This fact underlines the importance of developing new therapeutic strategies to address the widespread antibiotic resistance, which is the major contributing factor for clinical failures of the current therapeutics. In a screen for antibiotic adjuvants, we identified a natural product from actinomycetes, venturicidin A (VentA), that potentiates the aminoglycoside antibiotic gentamicin against multidrug-resistant clinical isolates of *Staphylococcus*, *Enterococcus*, and *Pseudomonas aeruginosa*. Furthermore, the combination of gentamicin and VentA was bactericidal and rapidly eradicated methicillin-resistant *S. aureus* (MRSA). The molecular mechanism of gentamicin potentiation activity is attributed to uncoupling of ATP synthesis by VentA from electron transport presumably by blocking the proton flow through ATP synthase, which results in an elevated concentration of extracellular protons and subsequent anticipated raise in gentamicin uptake. The disruption of the proton flux was characterized by perturbed membrane potential in MRSA. These results demonstrate that inhibition of ATP synthase along with the subsequent membrane dysregulation, as shown here with VentA, complements aminoglycoside antibiotics against MDR bacteria, and that this approach may be employed to combat bacterial resistance.

## INTRODUCTION

Multidrug resistance is now common in most bacterial pathogens^1^. Even so-called last resort drugs such as polymyxins, oxazolidinones, and carbapenems are increasingly inactive against many clinical, and sometime epidemic, strains^1–5^. Among the most concerning bacteria with a propensity toward multidrug resistance are the Gram-negative bacteria *Klebsiella pneumoniae*, *Acinetobacter baumannii*, *Pseudomonas aeruginosa* and the Gram-positive *Staphylococcus aureus*, especially methicillin-resistant (MRSA) strains^6^. The abandonment of antibiotic discovery and development by the majority of pharmaceutical companies and the consequent diminished drug pipeline is a grave global public health threat^7^.

One potential solution to the antibiotic crisis is the use of combinations of antibiotics, and antibiotic adjuvants^8^. The former are well established for the treatment of antibiotic tolerant bacterial pathogens such as enterococci and mycobacteria^9^ while the latter offer several strategies to enhance the activity of our existing antibiotic drugs, even against highly resistant strains^10^. Here we report the identification and characterization of a natural product adjuvant of the aminoglycoside antibiotic gentamicin.

Aminoglycosides were among the first antibiotics clinically deployed having broad coverage of bacterial spectrum and bactericidal activity^11^. They target the 30S ribosomal subunit, resulting in disruption of mRNA decoding with subsequent production of aberrant proteins^12,13^. They are primarily used for the treatment of infections caused by Gram-negative pathogens, but can also find use for Gram-positive bacteria, especially in combination with other antibiotics^11^. Bacterial resistance to aminoglycosides is common in many multidrug-resistant strains. The principal mechanisms of resistance include the expression of aminoglycoside-modifying enzymes (AMEs), efflux pumps, and enzymes that modify the 16S rRNA target by methylation^14,15^. Enzyme-catalyzed drug inactivation and ribosomal methylation are the most prevalent mechanisms of resistance in the clinic^15^. However, reduced antibiotic uptake, exemplified by efflux pumps in *P. aeruginosa*, and the generation of metabolically inactive small-colony variants common in staphylococci, are a growing concern^16,17^. The prevalence of AMEs informed the synthesis of next generation aminoglycosides, such as the recently approved antibiotic plazomicin, which is not a substrate for the most common inactivating enzymes^18^. Nevertheless, improving the activity of these potent drugs has the potential to address resistance in an orthogonal fashion^19–23^.

Antibiotic adjuvants are non-antibiotic molecules that enhance the potency of antibiotics against bacteria through direct blocking of resistance elements e.g. β-lactamase inhibitors (Type 1a adjuvants), or through indirect means (Type 1b adjuvants)^8,10^. Reasoning that the bacteria and fungi that produce most of our antibiotics may also be the source of adjuvants^24,25^, we have developed an in-house library of several thousand microbial extracts to screen for such activities. Using this resource, we screened for gentamicin rescue in an aminoglycoside resistant MRSA strain and identified venturicidin A (VentA) produced by a soil-isolated actinomycetes, WAC 9126, as an aminoglycoside adjuvant.

## Results

### Identification of venturicidin A as an aminoglycoside adjuvant

**Figure 1:**
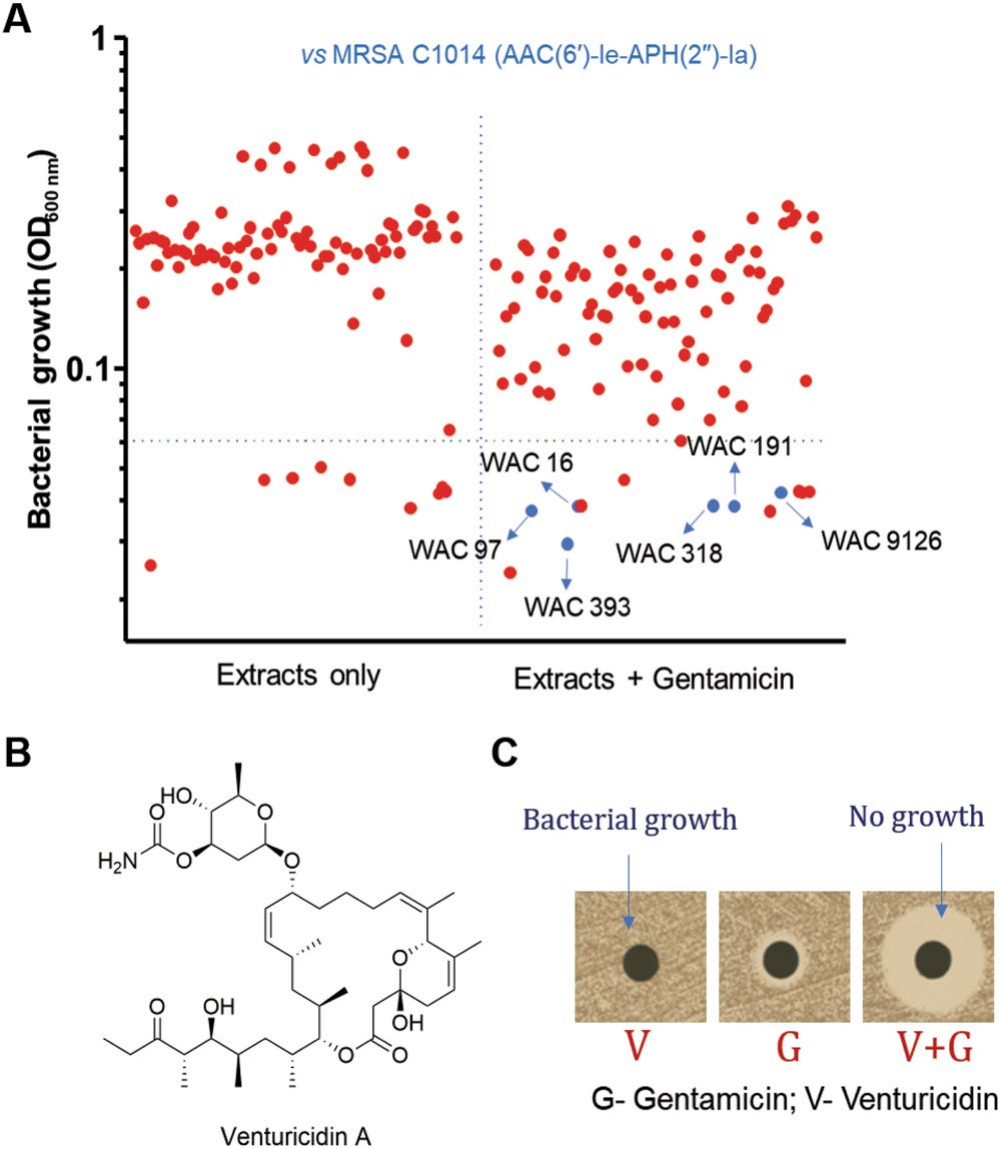
Screen for aminoglycoside adjuvants. (**A**) A primary screen of actinomycetes extracts for the potentiation of gentamicin against aminoglycoside-resistant MRSA C1014 harboring the bifunctional aminoglycoside-modifying enzyme, AAC(6′)-Ie-APH(2′′)-Ia. Blue color circles represent the extracts that potentiate gentamicin. (**B**) Chemical structure of venturicidin A isolated from WAC 9126 through activity-guided purification. (**C**) A Kirby-Bauer disk diffusion assay showing the effect of purified venturicidin A on the activity of gentamicin against MRSA C1014. 16 μg of venturicidin A and/or 16 μg of gentamicin on the disk. (G – gentamicin; V – venturicidin A; G + V – combination of gentamicin and venturicidin A). Clear zone represents the inhibition of bacterial growth.

We screened a library of actinomycetes extracts for the ability to rescue the activity of gentamicin against gentamicin-resistant MRSA that harbors a bifunctional AME, AAC(6′)-Ie-APH(2′′)-Ia. This bifunctional AME inactivates the antibiotic through acetylation and phosphorylation. We identified six extracts with the desired activity that do not inhibit bacterial growth on their own (Fig. 1A). Of these six extracts from the primary screen, one from strain WAC 9126 demonstrated significant and reproducible potentiation of gentamicin activity. This extract also showed antibacterial activity against a more sensitive bacterium, *Micrococcus luteus*, therefore *M. luteus* was used as a reporter organism for activity-guided purification. Fractionation of the methanolic extract by reversed-phase chromatography followed by silica gel chromatography of the partially pure active fractions yielded the pure compound (Supplementary Figs. S1 and S2). Structural elucidation of the active compound by multidimensional NMR spectroscopy (Supplementary Figs. S3–S7 and Table S1) and mass spectrometry (Supplementary Figs. S8 and S9) revealed the aminoglycoside adjuvant to be venturicidin A (VentA), a *Streptomyces*-derived molecule discovered in 1961 with antifungal activity^26,27^ (Fig. 1B). The antifungal mechanism of VentA was explored in the 1980s identifying it as an inhibitor of ATP synthase that acts by blocking the proton channel^28,29^. Gentamicin-rescue activity was confirmed by Kirby-Bauer disk diffusion assay with the pure VentA (Fig. 1C).

### Venturicidin A potentiates aminoglycosides against multidrug-resistant bacterial pathogens

**Figure 2:**
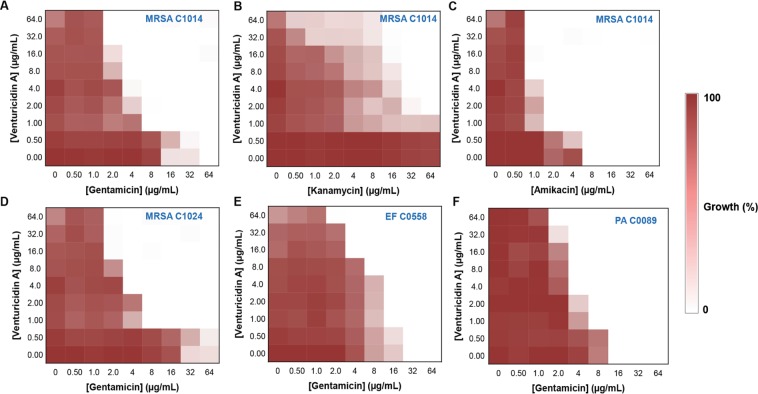
VentA overcomes aminoglycoside resistance in MDR clinical isolates. Checkerboard broth microdilution assays showing dose-dependent potentiation of aminoglycoside antibiotic by VentA. (**A,B,C**) Represent the ability of venturicidin A to potentiate gentamicin, kanamycin and amikacin respectively against MRSA C1014. (**D,E,F**) Represent the broad-spectrum activity of the combination against MDR isolates, MRSA C1024, EF C0558 and PA C0089, respectively. Dark regions represent higher cell density. For staphylococci and enterococci; the 100% growth corresponds to an OD_600_ of ~0.9 whereas for *Pseudomonas* it is ~0.4.

Purified VentA showed no antibiotic activity against a panel of ESKAPE bacterial pathogens (MIC > 256 µg/mL, Supplementary Table S2). VentA did show some growth impairment of some MRSA strains but none were completely inhibited (Supplementary Fig. S10). This growth phenotype was presumably in dormancy due to the expected effect of VentA on bacterial energy pathways. Next, the aminoglycoside-potentiation profiles of VentA was assessed in combination with gentamicin against a variety of aminoglycoside-resistant clinical isolates including MRSA, vancomycin-resistant enterococci (VRE), *P. aeruginosa*, *A. baumannii*, *E. coli* and *K. pneumoniae* (Table 1 & Fig. 2). Genome sequencing of these MDR clinical isolates revealed their mechanisms of aminoglycoside resistance^30^, and were due to the presence of efflux pumps, the bifunctional AME AAC(6′)-Ie-APH(2′′)-Ia, and the monofunctional AMEs, APH(3′)-IIIa, APH(3′)-IIb, APH(6)-1d, AAD(6), and AAC(6′)-Ii, and the ribosomal methyltransferase ArmA (Supplementary Table S3). All the MRSA isolates express the bifunctional AAC(6′)-Ie-APH(2′′)-Ia whereas VRE strains harbor both monofunctional AMEs (AAC, APH and AAD) and the bifunctional enzyme. The Gram-negative *Pseudomonas*, *Acinetobacter*, *E. coli* and *Klebsiella* isolates carry efflux mediated resistance genes and APH monofunctional AMEs. *Klebsiella* and *E. coli* also harbor AAC monofunctional AME. Two isolates of *A. baumannii*, C0074 and C0286 also encode an ArmA ribosomal methyltransferase. Our results demonstrate that VentA was able to potentiate the activity of gentamicin against the clinical isolates irrespective of the presence of AMEs or efflux pumps but did not show potentiation in the presence of ArmA that provides target protection (Table 1 and Supplementary Table S3).

**Table 1:** Antibacterial efficacy of gentamicin and VentA.

Bacteria	Minimum Inhibitory Concentration (µg/mL)			Venturicidin A
	Gentamicin			
	− Venturicidin A	+ Venturicidin A (8 µg/mL)	+ Venturicidin A (16 µg/mL)	
*MRSA C1014*	64	4 (0.125)	4 (0.187)	>128
*MRSA C1139*	32	4 (0.187)	2 (0.187)	>128
*MRSA C1024*	64	4 (0.125)	4 (0.156)	>128
*MRSA C0115*	16	2 (0.187)	2 (0.250)	>128
*CMRSA-3*	2	1 (1.062)	1 (1.125)	>128
*M. smegmatis*	0.25	0.125 (0.58)	0.125 (0.64)	>128
*E. faecium C0558*	32	8 (0.312)	4 (0.250)	>128
*E. faecium C0516*	16	4 (0.312)	4 (0.375)	>128
*P. aeruginosa C0060*	32	4 (0.187)	4 (0.250)	>128
*P. aeruginosa C0007*	8	8 (1.062)	4 (0.625)	>128
*P. aeruginosa C0089*	16	4 (0.312)	4 (0.375)	>128
*A. baumannii C0412*	8	2 (0.312)	2 (0.375)	>128
*A. baumannii C0074*	>64	>64	>64	>128
*A. baumannii C0286*	>64	>64	>64	>128
*E. coli BW25113*	2	1 (1.062)	1 (1.125)	>128
*E. coli C0001*	>64	>64	>64	>128
*K. pneumoniae C0108*	>64	64 (0.56)	64 (0.62)	>128

The values illustrated in parenthesis indicate the fractional inhibitory concentration index (FICI), measure of synergy (threshold ≤0.5). Though VentA does not have an MIC_90_, 128 µg/mL used as its lower limit MIC for FICI calculation.

Aminoglycoside-resistant MRSA clinical isolates C1014, C1024 and C1139 were highly resistant to gentamicin (MIC = 32 μg/mL–64 μg/mL) but when tested in combination with VentA, the MIC of gentamicin dropped 8 to 16-fold (Fig. 2A and Table 1), indicating significant potentiation of gentamicin activity. This potentiation was retained in the presence of the aminoglycoside antibiotics kanamycin, tobramycin and amikacin and is therefore not specific to gentamicin, but to the antibiotic class (Fig. 2A–D and Supplementary Fig. S11). Against both the gentamicin-resistant VRE isolates C0558 and C0516, the gentamicin activity was enhanced by 4 to 8-fold in presence of VentA (Fig. 2E and Table 1), a pattern conserved with *Pseudomonas, E. coli* and *Acinetobacter* isolates (Fig. 2F, Supplementary Fig. S11 and Table 1). In case of highly resistant *Klebsiella* isolate, C0108 and *E. coli* isolate, C0001, only a marginal or no gentamicin potentiation was observed. As aminoglycosides have been pivotal in the treatment of mycobacterial infections, next, the activity of the combination was tested against *M. smegmatis* and VentA potentiated the activity of gentamicin by 2-fold (Table 1). These results emphasize the broad-spectrum synergistic activity of the VentA-aminoglycoside combination (fractional inhibitory concentration index <0.5) against a variety of MDR clinical isolates.

### Venturicidin A specifically potentiates aminoglycosides

To test whether VentA also rescues the activity of other classes of antibiotics, potentiation activity was evaluated in combination with various antibiotics using the multi-drug resistant clinical isolate, MRSA C1014. VentA had no effect on amoxicillin, ciprofloxacin, or tetracycline MICs (Supplementary Fig. S11). VentA did overcome the intrinsic resistance of staphylococci towards polymyxins and the MIC of polymyxin B was found to be 4 μg/mL in presence of 16 μg/mL of VentA (Supplementary Fig. S11). Although *S. aureus* lacks lipopolysaccharide, the principal target of polymyxin, we speculate that VentA deenergizes the bacterial cell membrane, a known secondary target of polymyxins, and thereby facilitates interaction with the cationic polymyxin B. Recently, Vestergaard *et al*. reported a similar observation where deletion of non-essential genes for viability but required for ATP synthesis rendered staphylococci susceptible to polymyxins^31^.

### The combination of venturicidin A and gentamicin is rapidly bactericidal

**Figure 3:**
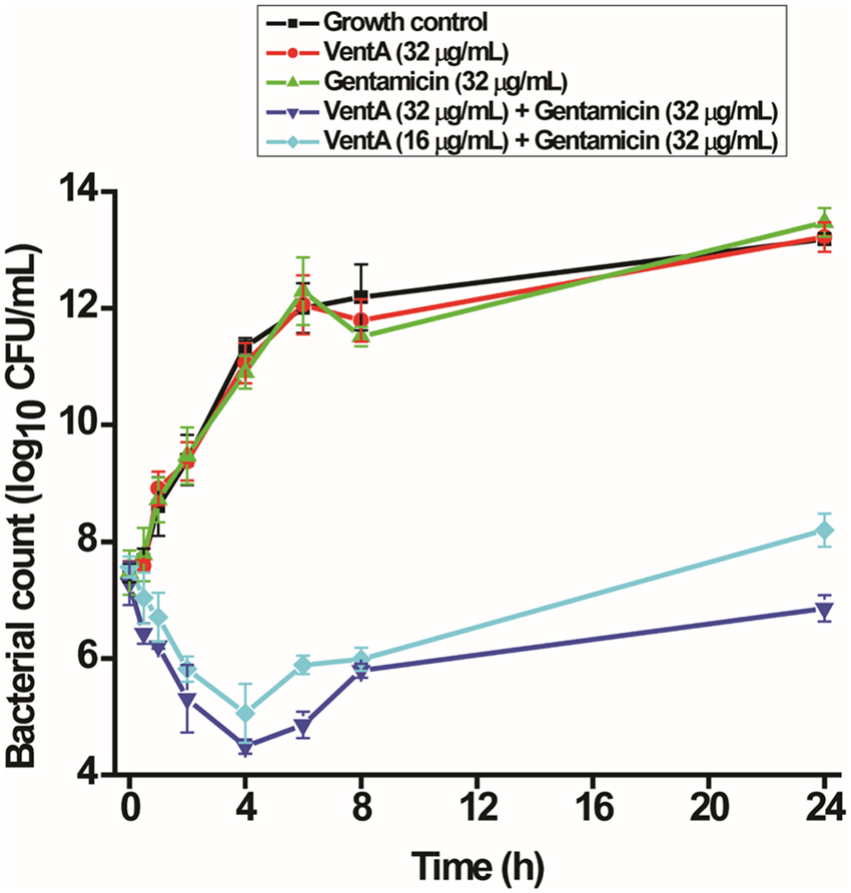
Time-kill kinetics of gentamicin in combination with VentA against MRSA C1014. The combination was rapidly bactericidal reducing the bacterial titer until 4 h and then a slight resuscitation of bacterial growth was observed whereas gentamicin and VentA alone were completely ineffective. The data are presented as mean ± standard deviation.

As aminoglycosides are known to be bactericidal, we carried out *in vitro* time-kill assays of the combination against the aminoglycoside-resistant MRSA C1014. The combination, unlike the antibiotic alone, displayed an impressively rapid bactericidal activity until 4 h producing >3 log_10_ CFU/mL reduction in viable cells (Fig. 3). But after 4 h a slight resuscitation of bacterial growth was observed with the combination probably due to the formation of antibiotic-tolerant variants, a phenomenon that generally occurs with aminoglycosides^32^. Therefore, VentA complements gentamicin in not only growth inhibition but also in killing of drug-resistant bacteria.

### Venturicidin A does not inhibit aminoglycoside-modifying enzymes

**Figure 4:**
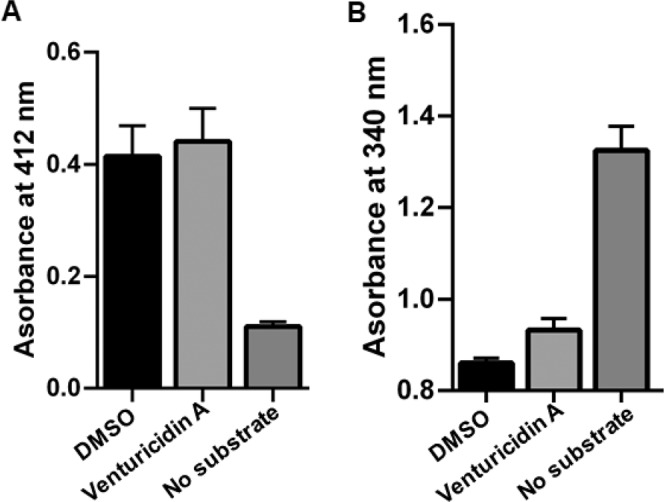
Effect of VentA on the action of bifunctional aminoglycoside-modifying enzyme, AAC(6′)-Ie-APH(2′′)-Ia. (**A**) AAC activity of bifunctional AME and (**B**) APH activity of bifunctional AME. VentA does not inhibit either of the enzymatic functions at the highest concentration tested, 112 μg/mL. The data are presented as mean ± standard deviation.

Resistance to aminoglycosides is often the result of drug modification catalyzed by AMEs. All Gram-positive cocci used in this study express the bifunctional AME, AAC(6′)-Ie-APH(2′′)-Ia. We tested the possibility that VentA was an AME inhibitor using the purified bifunctional enzyme in both kinase (APH) and acetyltransferase (AAC) modes. VentA showed no AME inhibition at concentration as high as 112 μg/mL (Fig. 4A,B). VentA therefore potentiates aminoglycosides through indirect means and is categorized as Type 1b adjuvant^10^.

### Venturicidin A depletes intracellular ATP levels of the bacteria

**Figure 5:**
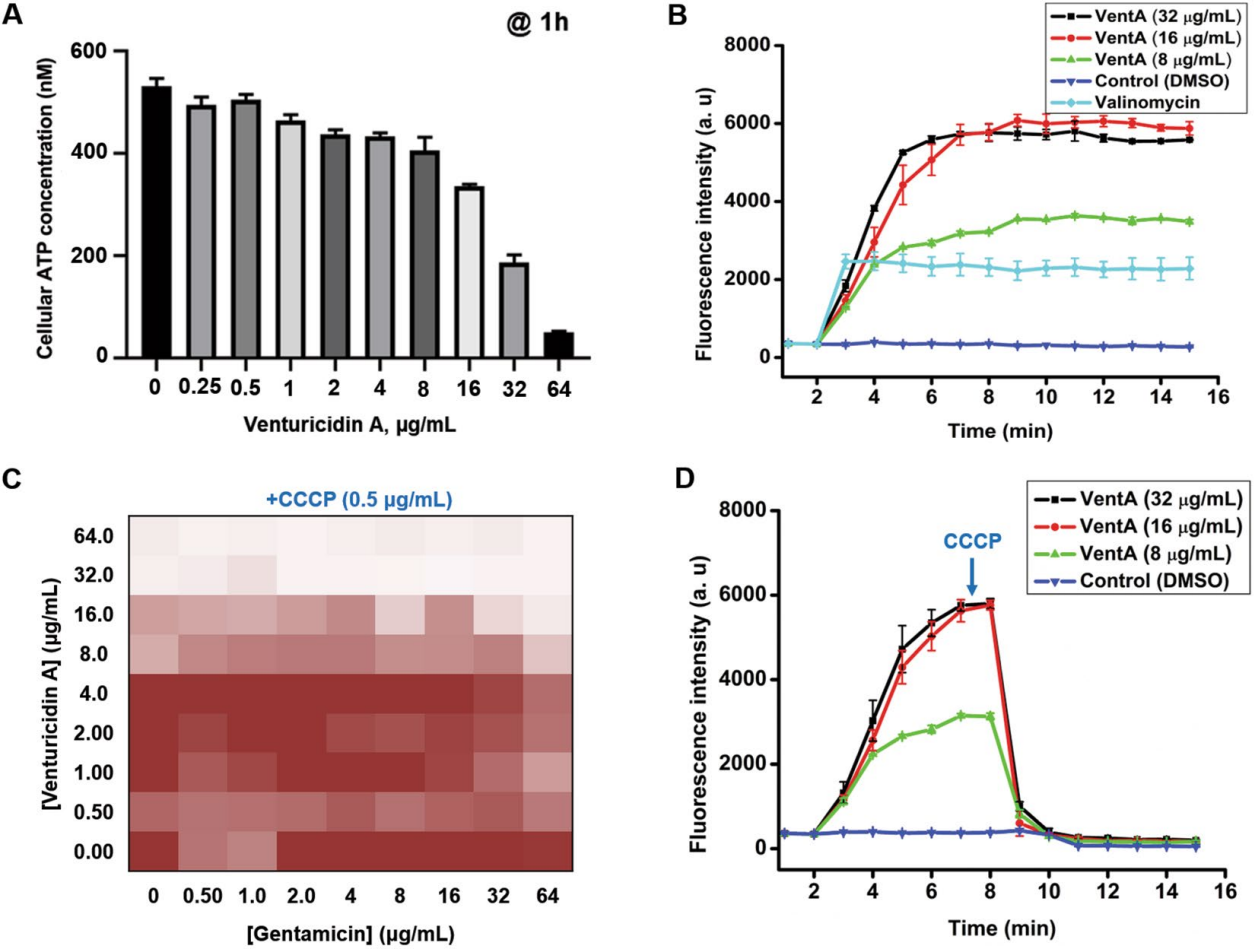
Mechanistic investigation of VentA. (**A**) Depletion of cellular ATP levels of MRSA C1014 by VentA in dose-dependent manner. (**B**) Cytoplasmic membrane depolarization ability of VentA against MRSA C1014 showing the concentration-dependent bacterial membrane depolarization. Valinomycin was used as a positive control at 20 µg/mL. (**C**) Effect of protonophore (CCCP) on the ability of VentA to potentiate gentamicin against MRSA C1014. CCCP antagonizes the VentA effect. (**D**) Effect of CCCP (2 µg/mL) on the membrane active nature of VentA measured by DiSC_3_(5) dye. The data are presented as mean ± standard deviation.

VentA inhibits the fungal ATP synthase by specifically blocking proton translocation through the F_O_ subunit^29^ and there are few reports on its effect on bacterial ATP synthesis^28,33^. We hypothesized that VentA may also inhibit the structurally and functionally homologous bacterial ATP synthase accounting for its aminoglycoside Type Ib adjuvant activity. VentA showed concentration-dependent depletion of cellular ATP in MRSA and dropped the ATP levels by approximately 10-fold compared to untreated controls (Fig. 5A). A similar ATP reduction in the presence of VentA was reported in *P. aeruginosa* by Armitage *et al*.^33^. VentA is therefore an inhibitor of ATP synthesis in fungi and bacteria.

### Venturicidin A depolarizes the bacterial membrane

Given the impact of VentA on the depletion of ATP levels, we envisioned that it may also dissipate the cell membrane potential. We evaluated depolarization of the MRSA C1014 membrane using the membrane potential sensitive dye, DiSC_3_(5) (3,3′-dipropylthiadicarbocyanine iodide). This hydrophobic cationic dye initially accumulates in the negatively charged bacterial membrane, which quenches its fluorescence at 670 nm. Disruption of the membrane potential results in an increase in fluorescence intensity due to the displacement of the dye from the membrane interior to the solution. VentA caused a dose-dependent rise in fluorescence intensity of the dye, and at 8 μg/mL was as effective as the positive control, valinomycin at 20 μg/mL (Fig. 5B). This observation is consistent with a mechanism where VentA-mediated blocking of proton translocation by ATP synthase increases the net concentration of extracellular protons at the membrane. This drives the leakage of the accumulated dye into the solution as a result of disruption in membrane potential.

### **The protonophore**, carbonyl **cyanide 3-chlorophenylhydrazine (CCCP), antagonizes venturicidin A effect**

Since VentA alters the membrane potential, likely by causing the accumulation of extracellular protons, we predicted that the proton ionophore CCCP, would oppose the VentA activity. In MRSA C1014 the activity of VentA alone is slightly enhanced whereas the activity of gentamicin alone is diminished in presence of CCCP (0.5 μg/mL). On the other hand, CCCP antagonized the VentA potentiation of gentamicin, and the combination was ineffective against MRSA C1014 (Figs. 2A and 5C). Next, to support our observation, we introduced CCCP to VentA-treated cells and measured the membrane potential using the DiSC_3_(5) dye. The addition of CCCP reversed the VentA effect presumably by abstracting the accumulated extracellular protons and caused considerable collapse in fluorescence intensity of the dye (Fig. 5D). It is important to mention that interference of CCCP with the dye fluorescence is minimal at the tested concentration (2 µg/mL) (Supplementary Fig. S12), and a significant reduction in fluorescence intensity is noticed in presence of bacterial cells indicating the protonophore effect of CCCP confers for maximal quenching in fluorescence. These results are consistent with the hypothesis that the accumulated extracellular protons due to the activity of VentA are linked to gentamicin potentiation and enabling the uptake of gentamicin. Although the detailed mechanism of aminoglycoside uptake is unclear, it is well established that a threshold proton-motive force (PMF) is required for the entry of aminoglycosides into the cell^34^.

### Venturicidin A enables intracellular accumulation of gentamicin

**Figure 6:**
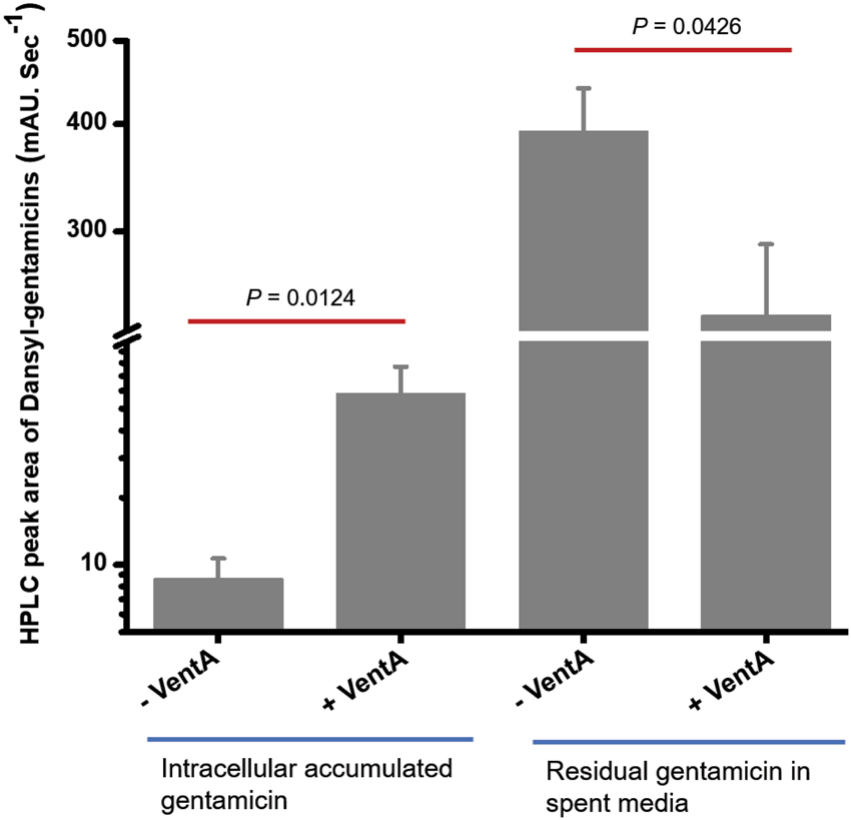
VentA enables intracellular uptake of gentamicin. Intracellular accumulated gentamicin in *A. baumannii* C0286 is significantly higher and residual gentamicin in spent medium is significantly lower in presence of VentA. The data are presented as mean ± standard deviation. Statistical significance is determined by using a two-sample Student’s *t*-test. Differences are considered statistically significant with a probability of *P* < 0.05.

To determine whether VentA enables gentamicin uptake, we measured the levels of intracellular accumulated antibiotic using pre-column derivatization with dansyl chloride and analyzed by liquid chromatography and mass spectrometry. On pre-column derivatization, the amine groups of gentamicin are dansylated and this facilitates for rapid identification of gentamicin. During dansylation, we observed tri-, tetra-, and penta-dansylated gentamicins (Supplementary Figs. S13–S15). The liquid chromatography peak for tri-dansylated gentamicin is overlapped with byproducts of the reaction and hence peaks corresponding to tetra- and penta-dansyl derivatives have been taken into consideration for measuring the gentamicin levels (Supplementary Fig. S13). Our assay for gentamicin uptake is not amenable with bacteria that harbor antibiotic-modifying enzymes because of the possible alteration of amine functional groups by AMEs. Therefore, we used *A. baumannii* C0286 as a proxy that harbors ArmA. We found that VentA facilitates intracellular accumulation of gentamicin and the levels are higher compared to only gentamicin-treated samples (Fig. 6). To validate this finding, we next measured the residual gentamicin present in spent medium. Our data demonstrate that the levels of residual gentamicin is significantly lower in presence of VentA (Fig. 6). These findings suggest that VentA potentiates aminoglycosides by facilitating intracellular accumulation.

Aminoglycosides are known to interact with the bacterial membrane and this property might enhance the intracellular uptake of VentA and reach unraveled targets. In order to see if gentamicin has any role on VentA cellular internalization, we have measured the intracellular levels of VentA in presence of gentamicin in *A. baumannii* C0286 cell lysates by liquid chromatography. There were no significant differences on the accumulation of VentA between cell lysates of gentamicin treated and untreated (Supplementary Fig. S16). This confirms that VentA cellular internalization is not influenced by the presence of gentamicin. However, it has the ability to permeabilize the bacterial cell membrane by itself and stalls the proton translocation across the ATP synthase and potentiates aminoglycoside antibiotics but lacks intrinsic antibiotic activity.

### Toxicology of venturicidinA

To evaluate the safety profile of the present aminoglycoside potentiator, we studied the *in vitro* toxicity of VentA toward mammalian cells; human embryonic-kidney (HEK) cells. VentA showed noticeable toxicity toward HEK cells with an IC_50_ of 31 μg/mL which is ~4-fold higher than what was required for gentamicin potentiation. In spite of having toxicity *in vitro*, the systemic toxicity data of VentA in mouse models is quite promising and it was reported to be 400 mg/kg (Intraperitoneal) and 20 mg/kg (Intravenous), demonstrating the high tolerability of the compound in animals when administered intraperitoneally^35^.

## Discussion

Antibiotic adjuvants rescue the activity of existing drugs and prolong their lifespan for clinical use; a promising way to mitigate the gap between the need for new drugs and the diminishing supply pipeline^8–10^. Several classes of antibiotics including β-lactams^8^, tetracyclines^36,37^, aminoglycosides^19–23^, rifamycins^38^ and polymyxins^39,40^ have been shown to be potentiated by antibiotic adjuvants against resistant-bacteria, and this encouraged our efforts to identify novel untargeted antibiotic adjuvants.

**Figure 7:**
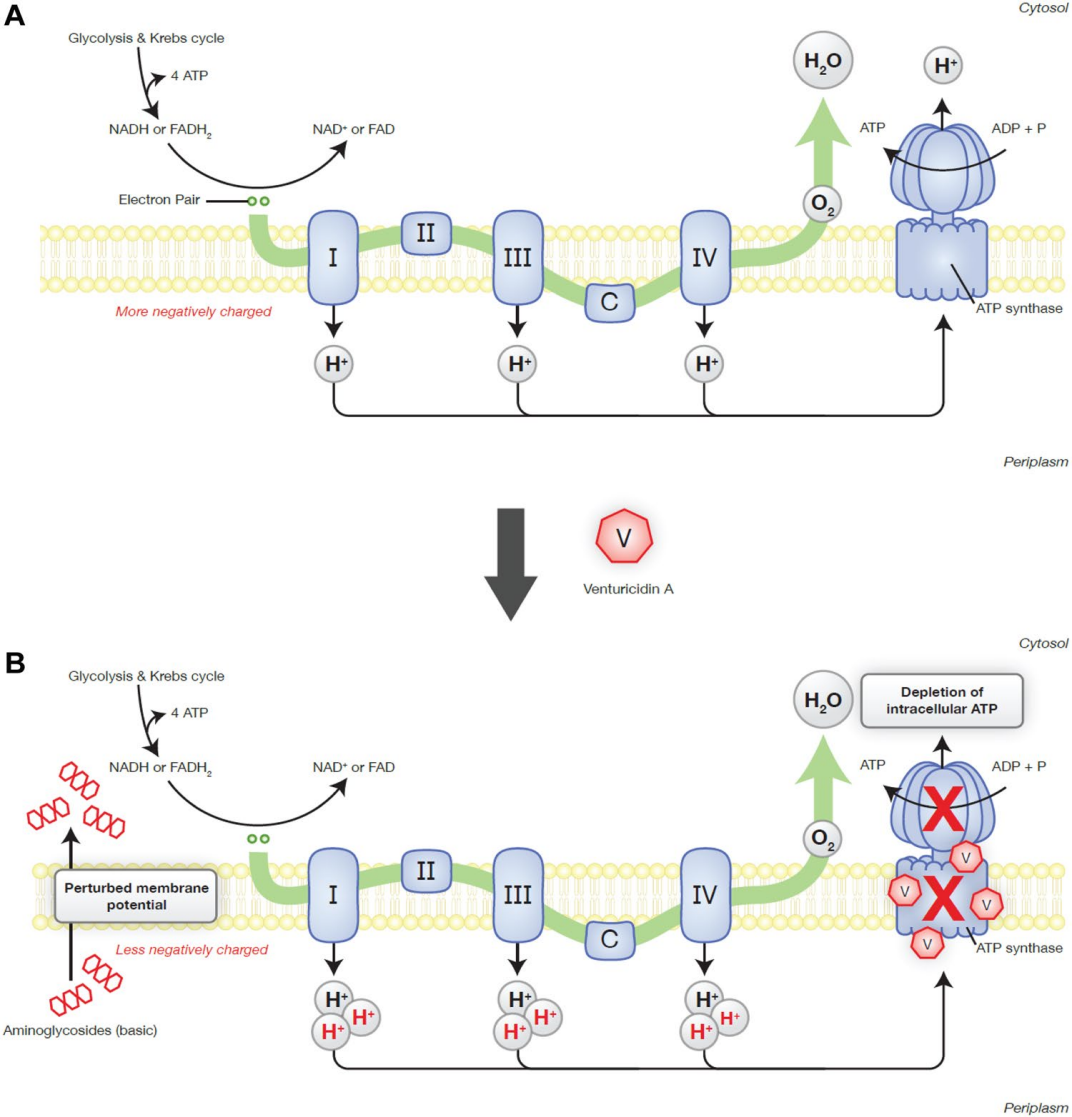
Proposed mechanism for VentA-enabled potentiation of aminoglycosides. (**A**) General mechanism of oxidative phosphorylation in bacteria. (**B**) Impact of VentA on oxidative phosphorylation. VentA blocks the proton translocation by the ATP synthase, and inhibits the ATP synthesis. Thus, the protons that are continuously generated in electron-transport chain accumulate extracellularly, cause imbalance in proton flux and dissipation in membrane potential. These accumulated extracellular protons are linked to gentamicin potentiation as the addition of a protonophore reversed the VentA-effect. Collectively, VentA-driven alterations at the membrane level promote the uptake of aminoglycoside antibiotic.

Since the majority of antibiotics originate from soil microbes, we envisioned that microbes could be also a potential source for antibiotic adjuvant discovery^24,25,40^. We screened a library of in-house actinomycetes extracts for aminoglycoside potentiation activity against resistant bacteria and identified venturicidin A (VentA), an inhibitor of ATP synthase, as a type 1b adjuvant. Studies in 1980s revealed that VentA binds to ‘c’ subunit of fungal ATP synthase and blocks proton translocation consequently inhibiting ATP synthesis^29^. As evidenced by our results, VentA also depletes the bacterial intracellular ATP levels, but it has little or no intrinsic antibacterial activity. However, VentA potentiates aminoglycosides against aminoglycoside-resistant clinical isolates of MRSA, VRE, *P. aeruginosa* and *A. baumannii*. VentA depolarizes the bacterial membrane by stalling the proton translocation, which results in dissipation of membrane potential and subsequent depletion of ATP levels. This further results in accumulation of extracellular protons, and imbalance in membrane potential which enhances aminoglycoside uptake (Fig. 7). This hypothesis is consistent with previously reported effects of the H^+^-ATPase inhibitor, *N*,*N’*-dicyclohexylcarbodiimide (DCCD), which also enhances the uptake of gentamicin^41^. But DCCD has been shown to hyperpolarize the bacterial membrane unlike VentA. DCCD is a covalent inhibitor of ATP synthase, conjugates to carboxylic acid of subunit ‘c’ by utilizing the protons of the carboxylic acid^42^. Considering the high reactivity of DCCD towards carboxylic acids in protonated state, it is surmised that any excess of DCCD across the membrane can react with a variety of carboxylic acids and forms a highly reactive intermediate, dicyclohexyl-*O*-acyl urea that can react with any available free amines. Thus, consumption of protons by DCCD and the possible reactivity of amines with the intermediate might lead to more negative charge across the membrane which results in hyperpolarization. On the other hand, VentA is specific to ATP synthase and it is expected to have non-covalent interactions like oligomycin and blocks the proton translocation which results in increased net positive charge^43^. Further, the lipophilic nature of VentA might facilitate its interaction with bacterial lipid membranes unlike DCCD. Therefore, we believe that the lipophilicity and enhanced net positive charge across the membrane confer membrane depolarization property to VentA. Thus, we envision that membrane perturbations caused by the treatments of DCCD and VentA might enable the enhanced uptake of aminoglycoside antibiotic. To further support our hypothesis, the protonophore, CCCP completely nullified the adjuvant activity of VentA and such compounds are known to reduce the intracellular uptake of aminoglycosides^41^.

Other ATP synthase inhibitors tomatidine and resveratrol have also been shown to potentiate aminoglycosides against Gram-positive pathogens especially *S. aureus*, however VentA appears to be slightly better than these comparators. Against aminoglycoside-resistant *S. aureus*, VentA (at 8 µg/mL) enhanced the activity of gentamicin up to a maximum of 16-fold, whereas resveratrol (at 128 µg/mL) and tomatidine (at 8 µg/mL) produced 4-fold and 8-fold, respectively^23,44^. Further β-lactam antibiotics^45^, arylomycins^46^, rifampicin^47^, *Cordia verbenacea* leaf extracts^48^, etc. produced either additive effect or slight synergy when tested in combination with gentamicin however the mechanism for this potentiation is unknown. More recently, *Pseudomonas*-derived rhamnolipids have been shown to potentiate tobramycin against antibiotic-tolerant staphylococci and the mechanism of potentiation is attributed to membrane active properties of rhamnolipids, which facilitated the intracellular accumulation of the antibiotic through a mechanism that is independent of the proton-motive force^49^. The significant ability of VentA to depolarize the bacterial membrane coupled with ATP synthase inhibition resulted in its impressive aminoglycoside potentiation.

Downstream pleiotropic effects due to depletion in cellular ATP levels might also contribute to aminoglycoside potentiation. Efflux pumps require an intact and energized cell membrane, hence VentA-mediated depletion of ATP levels and membrane perturbation likely indirectly impact pump mediated resistance^50^. We also note that AMEs are directly or indirectly ATP-dependent and can be affected by intracellular ATP levels. Thus, inhibition of ATP synthesis by blocking the proton translocation overcomes aminoglycoside antibiotic resistance and this study demonstrates the value of untargeted antibiotic adjuvant screens to discover potential new solutions to overcoming antibiotic resistance. VentA appears to be moderately safe aminoglycoside potentiator when administered intraperitoneally^35^. Nevertheless, identification of VentA biosynthetic gene cluster in the producer strain and performing rational biosynthetic manipulations on it are warranted in order to obtain a better analog that demonstrates greater specificity toward bacteria. Another interesting approach can be antibiotic hybrids where VentA can be chemically conjugated to aminoglycoside antibiotics through a labile bond with a linker, in such case the toxicity of the physical combination can be reduced while maintaining their synergistic antimicrobial profiles.

## Conclusions

Venturicidin A, a natural product isolated from actinomycetes (WAC 9126), represents a new Type Ib adjuvant for aminoglycosides and restores the activity of gentamicin against drug-resistant clinical isolates. While the inherent toxicity of VentA likely precludes it from further clinical use, the molecular mechanism of VentA reveals a general strategy to potentiate aminoglycosides via inhibition of ATP synthase with consequent dysregulation of proton translocation facilitating aminoglycoside entry into the cell. As combination therapeutic strategies are increasingly being considered as a means to extend the life of existing antibiotic drugs, the mechanism revealed by VentA is promising for further targeting to address the challenge of aminoglycoside resistance.

## Methods

### Materials and Instrumentation

A subset of the Wright Actinomycetes Collection (WAC), isolated from soil samples across Canada were used in this study. Multidrug-resistant bacterial isolates were procured from Hamilton Health Sciences, Canada. All media components and solvents were purchased from Fisher Scientific. BacTiter-Glo microbial cell viability assay kit from Promega was used to determine the ATP levels. Bennets’ media was used to culture *Streptomyces*. All strains except enterococci were grown in cationic adjusted Mueller – Hinton broth (CA-MHB) at 37 °C. Brain-heart infusion broth (BHI) was used to culture enterococci. Antibiotics gentamicin, kanamycin and tobramycin were purchased from Fisher Scientific; and amikacin, ampicillin, tetracycline, polymyxin B, ciprofloxacin, CCCP and valinomycin were purchased from Sigma. Membrane potential sensitive dye, DiSC_3_(5) (3,3′-dipropylthiadicarbocyanine iodide) and dansyl chloride were purchased from Sigma. Diaion HP-20 resin and deuterated solvents were obtained from Sigma.

Flash chromatography fractionation was performed on CombiFlash system, Teledyne ISCO. The liquid chromatography separation was performed on Agilent 1280 Infinity instrument. High-performance liquid chromatography (HPLC) coupled with MS detection (LC/MS) using a QTRAP LC/MS/MS System (Applied Biosystems) was used to analyze the active fractions. High-resolution mass spectra were recorded on Agilent 1290 Infinity II LC System (Agilent Technologies) and a qTOF 6550 mass detector. NMR data were acquired on Bruker AVIII 700 MHz instrument equipped with a cryoprobe. Molecular Devices Spectramax plus 384 plate reader was used to measure the bacterial growth. TECAN plate reader (Sunrise) was used to measure the growth curves. Luminescence and fluorescence were recorded on Biotek Synergy H1 plate reader.

### Primary screening of natural product library (NPL) extracts

The NPL is a collection of microbial fermentation extracts derived from strains within the WAC. A subset of NPL extracts were used to identify producers of adjuvants for aminoglycosides. Screening was performed in U-bottom, 96-well microtiter plates (Sarstedt, Canada) as described previously^25^ with slight modifications. Briefly, aminoglycoside-resistant MRSA C1014 was used to screen the NPL in the absence of and in combination with gentamicin at a concentration of 16 μg/mL (0.25 MIC). Bacterial growth was monitored by measuring the OD_600_ and the data was plotted to identify the extracts that only reduced the growth of MRSA C1014 in combination with gentamicin. We identified six hits in our primary screen that potentiated gentamicin. Then, during our efforts to verify the primary hits, we had significant difficulties with growing WAC 16. When tested the activity of freshly made crude methanolic extracts of the remaining five; WAC 191 itself had the activity. The extracts of WAC 97, WAC 318, WAC 393 and WAC 9126 retained gentamicin potentiation with differential levels of antibiotic rescue. WAC 97, WAC 393 and WAC 9126 extracts potentiated gentamicin significantly and reduced the MIC of gentamicin to 2 µg/mL–4 µg/mL whereas WAC 318 enabled moderate potentiation (8 µg/mL–16 µg/mL). Therefore, we fractionated WAC 97, WAC 393 and WAC 9126 crude extracts. The fractions of WAC 97 and WAC 393 lost the ability to potentiate gentamicin, presumably due to degradation or multiple compounds leading to potentiation. Conversely, a fraction from WAC 9126 maintained its gentamicin potentiation and hence this was chosen for further investigation.

### Activity-guided purification of aminoglycoside adjuvant from WAC 9126

For large scale production of VentA, WAC 9126 was streaked out on 6 plates containing Bennett’s agar (0.5 L of media on each plate) and incubated at 30 °C for 5 days (Supplementary Fig. S1). For extraction of metabolites, agar plugs were suspended in methanol and incubated overnight at 4 °C with shaking. The methanolic extract was filtered and concentrated to dryness by evaporation under reduced pressure. The crude material was redissolved in methanol and adsorbed on to HP-20 (Diaion) resin. The resin was loaded onto a column and eluted with H_2_O (1 L), 20% MeOH (1 L), 40% MeOH (1 L), 60% MeOH (1 L), 80% MeOH (1 L) and 100% MeOH (1 L), yielding six fractions. The VentA-sensitive organism, *Micrococcus luteus* was used as a reporter for activity-guided purification and the fractions, 80% MeOH and 100% MeOH maintaining the activity. The combined active fractions were subjected to reverse phase flash chromatography using a C_18_ column and a linear gradient of acetonitrile in water; and collected 41 fractions. Bioactive component was eluted in 95% to 100% acetonitrile; fractions 27 to 41. Next, these active fractions were combined and further purified by normal phase flash chromatography with a linear gradient of ethyl acetate in hexane. Active compound was eluted in 90% to 100% ethyl acetate, and the fractions were analyzed for purity using silica gel thin-layer chromatography (TLC) and reverse-phase HPLC. The analysis was performed on Agilent 1280 Infinity instrument using BDS Hypersil C_8_ column (4.6 × 250 mm, 5 μm) (Thermo Scientific) with mobile phase A (0.1% formic acid in water) and mobile phase B (0.1% formic acid in acetonitrile) monitoring the absorbance at 195 nm. The flow rate was 1 mL/min. The linear gradient was as follows: 0–1 min, 50% mobile phase A; 2–20 min, 50%–0% mobile phase A; 21–22 min, 50% mobile phase A. HPLC coupled high resolution mass spectral data was obtained using a C_18_ column (SunFire C_18_ 5 μm, 4.6 × 50 mm, Waters) at a flow rate of 0.4 mL/min, under the following conditions: isocratic 5% solvent B (0.1% formic acid in acetonitrile) and 95% solvent A (0.1% formic acid in water) for 1 min, followed by a linear gradient to 97% B over 10 min. The exact mass of VentA was 772.4606 [M + Na]^+^. The structure of the active compound was confirmed by 1D and 2D NMR (COSY, HSQC and HMBC) experimental data performed in deuterated dimethylsulfoxide (DMSO-D_6_). The acquired analytical data of the aminoglycoside adjuvant are in agreement with the known *Streptomyces*-derived natural product, venturicidin A^26,27^. In one of the active fractions, a small amount of venturicidin C was also observed; HR-ESI-MS: 786.4781 [M + Na]^+^.

### Drug-susceptibility assays

MIC tests and checkerboard assays were performed in U-bottom, 96-well microtiter plates using a modified broth microdilution protocol as described previously following CLSI guidelines^24,25,37^. In brief, assays were set up in a total volume of 0.1 mL/well with 2-fold dilutions of drug in CA-MHB or BHI. Plates were incubated at 37 °C for 24 h and the optical density was measured at 600 nm. At least 2 replicates were done for each combination. For growth curve analysis, overnight cultures were diluted to an optical density of ~0.05 at 600 nm (OD_600_) in CA-MHB, with or without the compound as indicated, and grown at 37 °C with continuous shaking using TECAN plate reader. OD_600_ was measured every 1 min over 24 h.

As a measure of synergy, fractional inhibitory concentration index (FICI) was calculated. FICI represents the sum of the FICs of each compound tested. The FIC for each drug is determined by dividing the MIC of each drug when used in combination by the MIC of each drug when used alone^10^. Though VentA was not active as an antibiotic, its MIC was considered as 128 µg/mL for FICI calculation.

### Bactericidal kinetics

MRSA C1014 grown in CA-MHB was used for time-kill kinetics. Experiments were performed in culture tubes in 3 mL volume using an initial inoculum concentration of ~4 × 10^7^ CFU/mL. Next, test compounds (Gentamicin, 32 µg/mL; VentA, 32 µg/mL; and the combination of VentA (16 µg/mL and 32 µg/mL) with gentamicin (32 µg/mL) were added to the wells at the concentrations as indicated and incubated at 37 °C. Aliquots (50 μL) of the bacterial suspension were removed at the specified time intervals (0, 0.5, 1, 2, 4, 6, 8 and 24 h) and 10-fold serial dilutions were performed in 0.85% saline, plated on CA-MHB agar plates, and incubated at 37 °C for 24 h. Then, the viable colonies were counted and represented as CFU/mL.

### Aminoglycoside modifying enzyme activity

Purified bifunctional AME, AAC(6′)-Ie-APH(2′′)-Ia was used to test possible VentA effects on enzymatic function. Both enzymatic assays (acetyltransferase and phosphotransferase activities) were performed following previously published protocols^18,51^ using VentA and gentamicin at 112 µg/mL. The acetylation of gentamicin was assessed by monitoring the absorbance at 412 nm which is expected to increase when 5,5′-dithiobis(2-nitrobenzoic acid) reacts with the CoA–SH that is derived from acetyl CoA after antibiotic acetylation. Phosphorylation of aminoglycoside substrate was followed using a pyruvate kinase/lactate dehydrogenase coupled assay system, which links the release of ADP to the oxidation of NADH to NAD^+^, and the resulting decrease in absorbance at 340 nm was measured.

### Determination of cellular ATP levels

The effect of VentA on cellular ATP levels of MRSA C1014 was determined using Promega BacTiter-Glo Microbial Cell Viability Assay kit following manufacturer’s instructions. The assay was performed in 96-well U-bottom plate in triplicate, and luminescence was recorded in white 96-well plates. MRSA was grown in CA-MHB at 37 °C overnight (OD_600_ ~ 0.6) and cells were harvested by centrifugation, washed and resuspended in fresh medium. Next, a 100 µL of 20-fold diluted bacterial culture was added to the wells of the 96-well plate, treated with VentA at different concentrations and incubated for 1 h at 37 °C. Next, 50 µL of bacterial suspension from each well was mixed with an equal volume of BacTiter-Glo Reagent and incubated for 5 min at room temperature. Luminescence was recorded on Biotek Synergy H1 plate reader. Signals represent the mean of three replicates for each measurement. The concentration of cellular ATP was estimated from the ATP standard curve.

### Membrane depolarization assay

The ability of VentA to disrupt the bacterial membrane was analyzed by using the fluorescent probe, 3,3′-dipropylthiacarbocyanine (DiSC_3_(5)) following previously described protocols with slight modification^25,37^. Briefly, MRSA C1014 was grown for 6 h in CA-MHB at 37 °C. Cells were harvested by centrifugation and washed in a buffer containing 250 mM sucrose, 5 mM MgSO_4_, and 10 mM potassium phosphate (pH 7.0). After three washings, cell pellets were resuspended in the same buffer and diluted the culture to OD_600_ ~ 0.1. The assay was performed in black 96-well plate in 100 µL volume in triplicate. The cells were incubated with DiSC_3_(5) at 1 µM for 20 min to quench the fluorescence of the dye by accumulating on bacterial membrane. Next, test compounds were added at different concentrations and measured the fluorescence for 15 min on Biotek Synergy H1 plate reader (Excitation wavelength – 622 nm and emission wavelength – 670 nm). Valinomycin at 20 µg/mL was used as a positive control.

To test the effect of protonophore on the depolarization ability of VentA; CCCP was added to the VentA-treated wells at 7 min and recorded the fluorescence of the dye further for 8 min.

### Intracellular drug accumulation assay

The accumulation assay was performed in triplicates with *A. baumannii* C0286 which lacks AMEs that modify gentamicin but harbors ArmA. The experiment was performed following previously described protocol^52^. Briefly, a freshly grown 250 mL bacterial culture in CA-MHB broth measuring OD_600_ of 0.5 was used for the experiment. The bacterial cells were collected, washed with 40 mL of phosphate buffered saline (PBS) and re-suspended in 10 mL of fresh PBS. The bacterial suspension was aliquoted into twelve Eppendorf tubes (800 μL each). The samples were equilibrated at 37 °C with shaking for 5 min, test compounds (control; gentamicin, 128 μg/mL; VentA, 64 μg/mL; and gentamicin, 128 μg/mL + VentA, 64 μg/mL) were added, and then samples were incubated at 37 °C with shaking for 10 min. Bacteria were pelleted by centrifuging at 13,000 r.c.f. for 2 min at room temperature. The residual supernatant was used to assess for remained gentamicin. Each cell pellet was dissolved in 200 μL of water and then lysed by subjecting the cells to three freeze-thaw cycles of three minutes in liquid nitrogen and in water bath at 65 °C. The lysates were pelleted at 13,000 r.c.f. for 3 min at room temperature and the supernatant was collected. The debris was re-suspended in 100 μL of water and pelleted as before. The supernatants were collected and combined with the previous cell lysate supernatants (a total of 300 µL of lysate for each sample). Finally, residual debris was removed by centrifuging at 15,000 r.c.f. for 15 min at room temperature.

#### Analysis of gentamicin by pre-column derivatization

Pre-column derivatization of gentamicin was performed with dansyl chloride, following previously described protocol with minor modifications^53^. Initially, to generate the standard sample, a 250 µL of 4 mg/mL gentamicin solution was mixed with alkalinized phosphate buffer (0.8 mL, pH 7.4, 66 mM, plus 30 µL of sodium hydroxide solution, 1.25 M). Then, 0.3 mL of acetonitrile containing 3 mg of dansyl chloride was added to it. Dansylation reaction was initiated by placing the reaction tube in water bath for 15 min at 65 °C and then cooled in ice bath. Next, 2 mL of ethyl acetate and 4 mL of carbonate buffer (0.5 M sodium bicarbonate and 0.5 M sodium carbonate, pH 9.5) were added to the cooled reaction mixture, which was vortex-mixed and centrifuged. The organic phase was collected, evaporated and dissolved in 200 µL of methanol. Then 10 µL of dansylated gentamicin solution was analyzed by HPLC and HR-MS. The liquid chromatography separation was performed on Agilent 1280 Infinity instrument using XTerra RP 18 column (4.6 × 150 mm, 5 μm) (Waters) with mobile phase A (0.1% formic acid in water) and mobile phase B (0.1% formic acid in acetonitrile) monitoring the absorbance at 254 nm and 345 nm with a flow rate of 1 mL/min. The linear gradient was as follows: 0–3 min, 95%–60% mobile phase A; 4–20 min, 60%–0% mobile phase A; 21–22 min, 0% mobile phase A; 23–25 min, 95% mobile phase A. Dansylated gentamicins were eluted at retention times 10.5 min (Tri-dansylation), 13.9 min (tetra-dansylation) and 18.6 & 18.8 min (penta-dansylation). HR-ESI-MS were acquired using an Agilent 1290 Liquid Chromatography and a qTOF 6550 mass detector in positive ion mode. The degree of dansylation was confirmed by HR-MS.

After standardizing the pre-column derivatization, 250 µL of cell lysates were analyzed for the presence of gentamicin as described above. Peak-area measurements of tetra- and penta-dansylated gentamicins were used for analysis. Tri-dansylated peak was overlapped with one of the reaction byproducts presumably dansyl acid hence it was not considered for analysis. Next, 800 µL of residual supernatant was used to assess remained gentamicin. In this case 220 µL of water and 30 µL of 1.25 M NaOH solution were added. Then the remaining steps were performed as mentioned above.

#### Analysis of VentA

A portion (30 µL) of cell lysate obtained in previous step was analyzed on HPLC to see if gentamicin has any role on accumulation of VentA. The analysis was performed on Agilent 1280 Infinity instrument using BDS Hypersil C_8_ column (4.6 × 250 mm, 5 μm) (Thermo Scientific) with mobile phase A (0.1% formic acid in water) and mobile phase B (0.1% formic acid in acetonitrile) monitoring the absorbance at 195 nm. The flow rate was 1 mL/min. The linear gradient was as follows: 0–4 min, 95%–50% mobile phase A; 5–24 min, 50%–0% mobile phase A; 25–26 min, 0% mobile phase A; 27–29 min, 95% mobile phase A. The standard sample of VentA eluted at 17.3 min. Peak-area measurements of VentA were used for analysis.

### Cytotoxicity assay

Human-embryonic kidney (HEK) cells were used to assess the toxicity of VentA. On day 1, HEK cells were seeded at 15,000 cells/well in 96-well tissue culture treated white plates in 100 μL of Dulbecco’s Modified Eagle’s Medium (DMEM) supplemented with 10% fetal bovine serum (FBS), and 2 mM L-glutamine. Cells were incubated for 18 h at 37 °C under 5% CO_2_ to reach confluency. After 18 h, the media was removed and fresh media containing VentA was added to the cells. VentA was solubilized in DMSO and the final DMSO concentration was 1%. Plates were incubated for 48 h and cell viability was assessed using Promega Cell Titer Glo reagent (Fisher Scientific). Then, 100 μL of Cell Titer Glo was added directly to the media, the plates were shaken for 2 minutes and then incubated for 10 minutes at room temperature. The luminescence was recorded on an EnVision plate reader (Perkin Elmer). IC_50_ curves were fitted using a four-parameter logistic (4PL) non-linear regression model.

The equation used for the 4PL curves was: 𝑦 = 𝑑+ [𝑎 − 𝑑/1 + (𝑥/𝑐)𝑏]

where, y = the sample response in relative luminescence units; x = the drug concentration; a = the maximum response for infinite standard concentration; b = -Hill slope; c = inflection point; d = the response at a standard concentration of 0

Using this equation, the IC_50_ values were calculated as 50% of the maximum response.

## Supplementary information

Supplementary information.
