# Supplementary material for: Venturicidin A, A Membrane-active Natural Product Inhibitor of ATP synthase Potentiates Aminoglycoside Antibiotics
Source: Sci Rep. 2020 May 18;10:8134. doi: 10.1038/s41598-020-64756-0 (PMC7235042; doi:10.1038/s41598-020-64756-0)
Supplement: Supplementary file 1 — Supplementary information. [file 41598_2020_64756_MOESM1_ESM.doc]

**Supplementary Data**

**Venturicidin A, A Membrane-active Natural Product Inhibitor of ATP synthase Potentiates Aminoglycoside Antibiotics**

Venkateswarlu Yarlagadda^1^, Ricardo Medina^2^, Gerard D. Wright^1,*^

^1^David Braley Centre for Antibiotic Discovery, M.G. DeGroote Institute for Infectious Disease Research, Department of Biochemistry and Biomedical Sciences, DeGroote School of Medicine, McMaster University, 1280 Main Street West, Hamilton, Ontario L8S 4K1, Canada

^2^Department of Microbiology, Central University of Las Villas, Santa Clara, Villa Clara, Cuba

**Table of Contents**

Page S2-S16: Supplementary Figures

Page S17-S19**:** Supplementary Tables

**I. Supplementary Figures**

**Figure S1.** Schematic representation of workflow for the activity-guided purification of venturicidin A from WAC 9126

**Figure S2.** The HPLC chromatogram of venturicidin A.

**
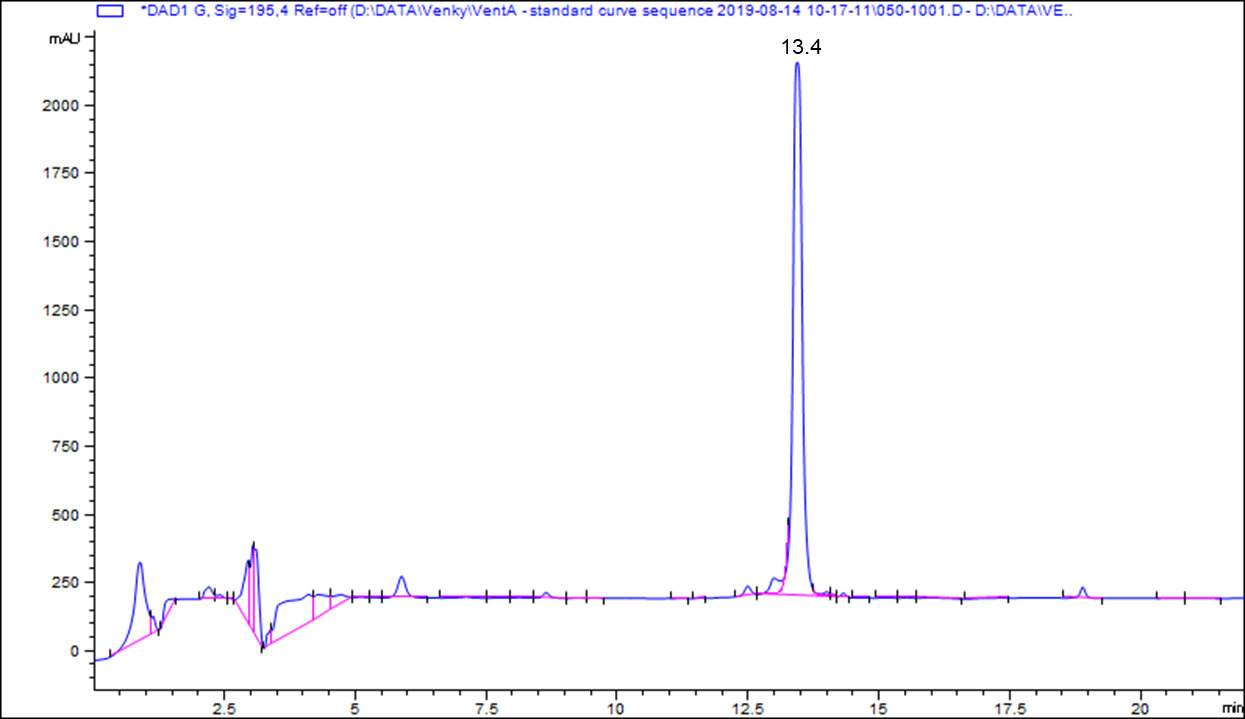
**

**Figure S3.** The ^1^H-NMR spectra of venturicidin A


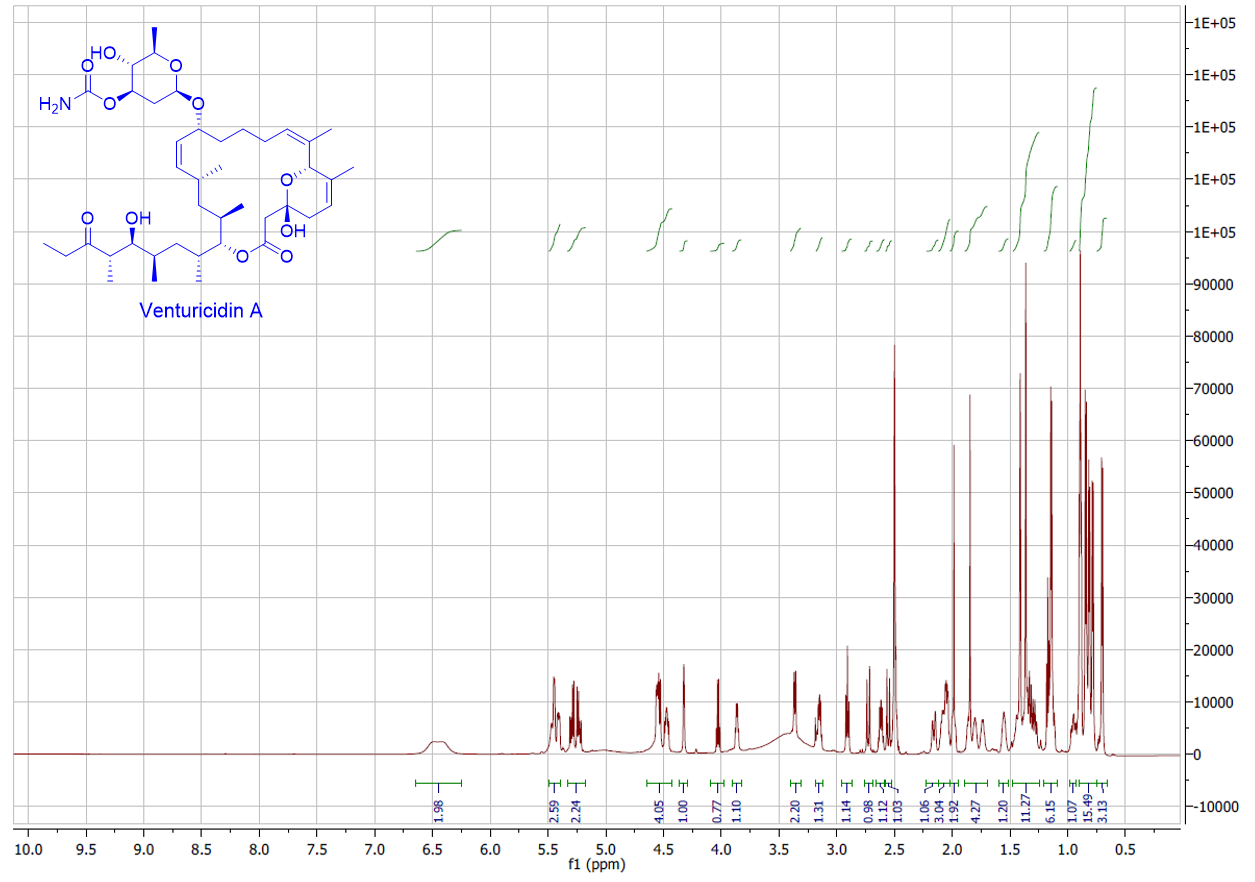


**Figure S4.** The ^13^C-NMR (DEPTQ) spectra of venturicidin A


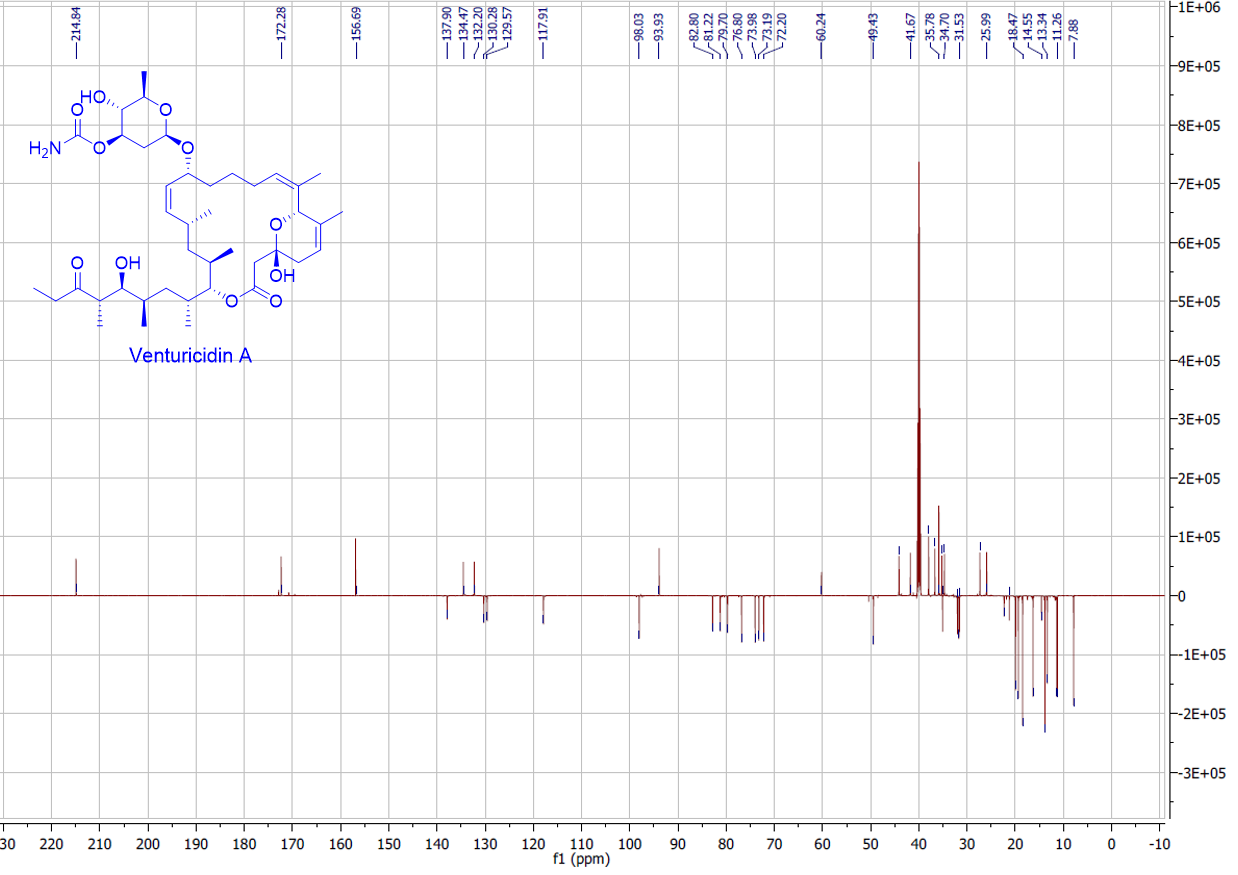


**Figure S5.** The ^1^H-^1^H COSY NMR spectra of venturicidin A


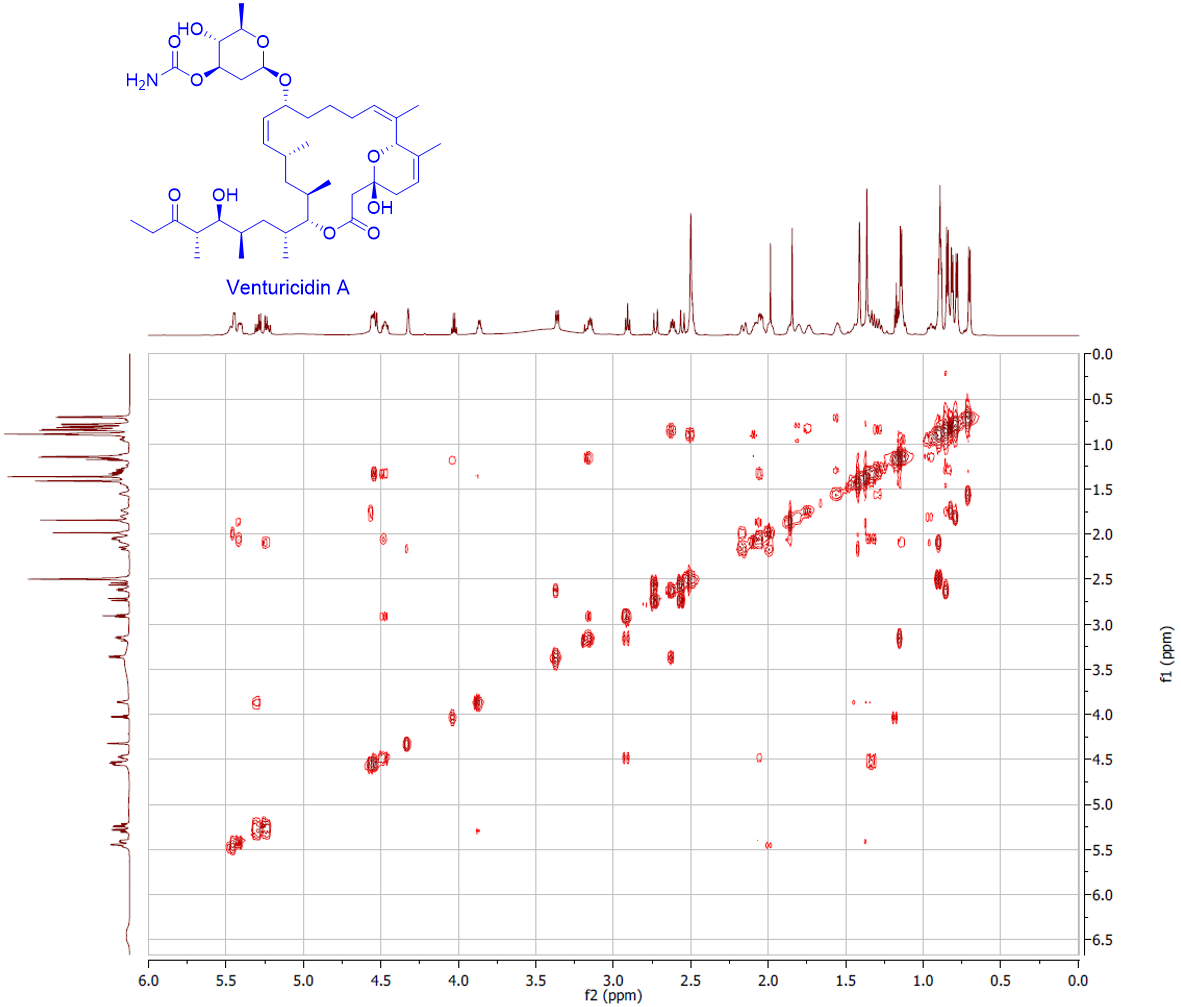


**Figure S6.** The ^1^H-^13^C HSQC NMR spectra of venturicidin A


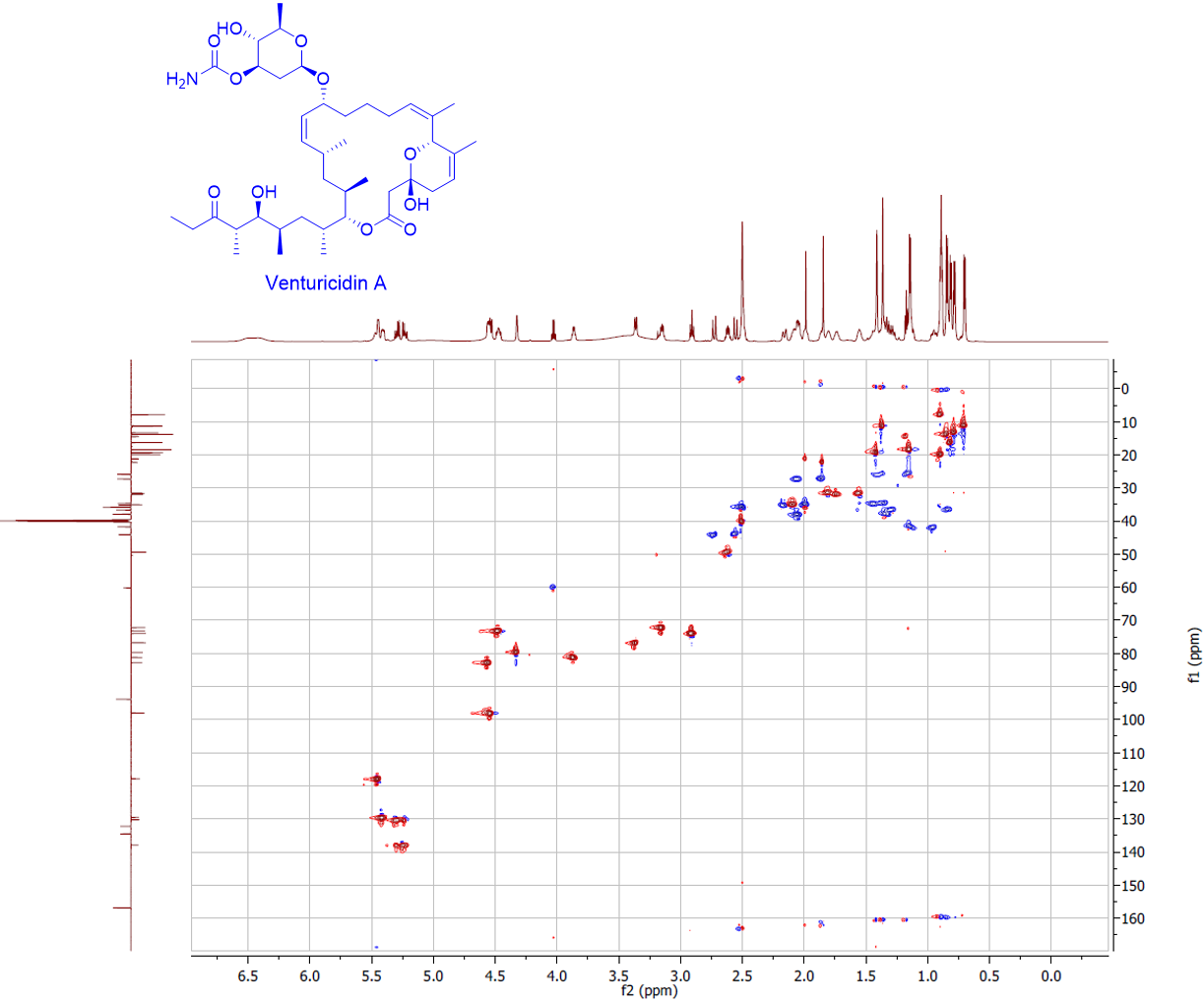


**Figure S7.** The ^1^H-^13^C HMBC NMR spectra of venturicidin A

**
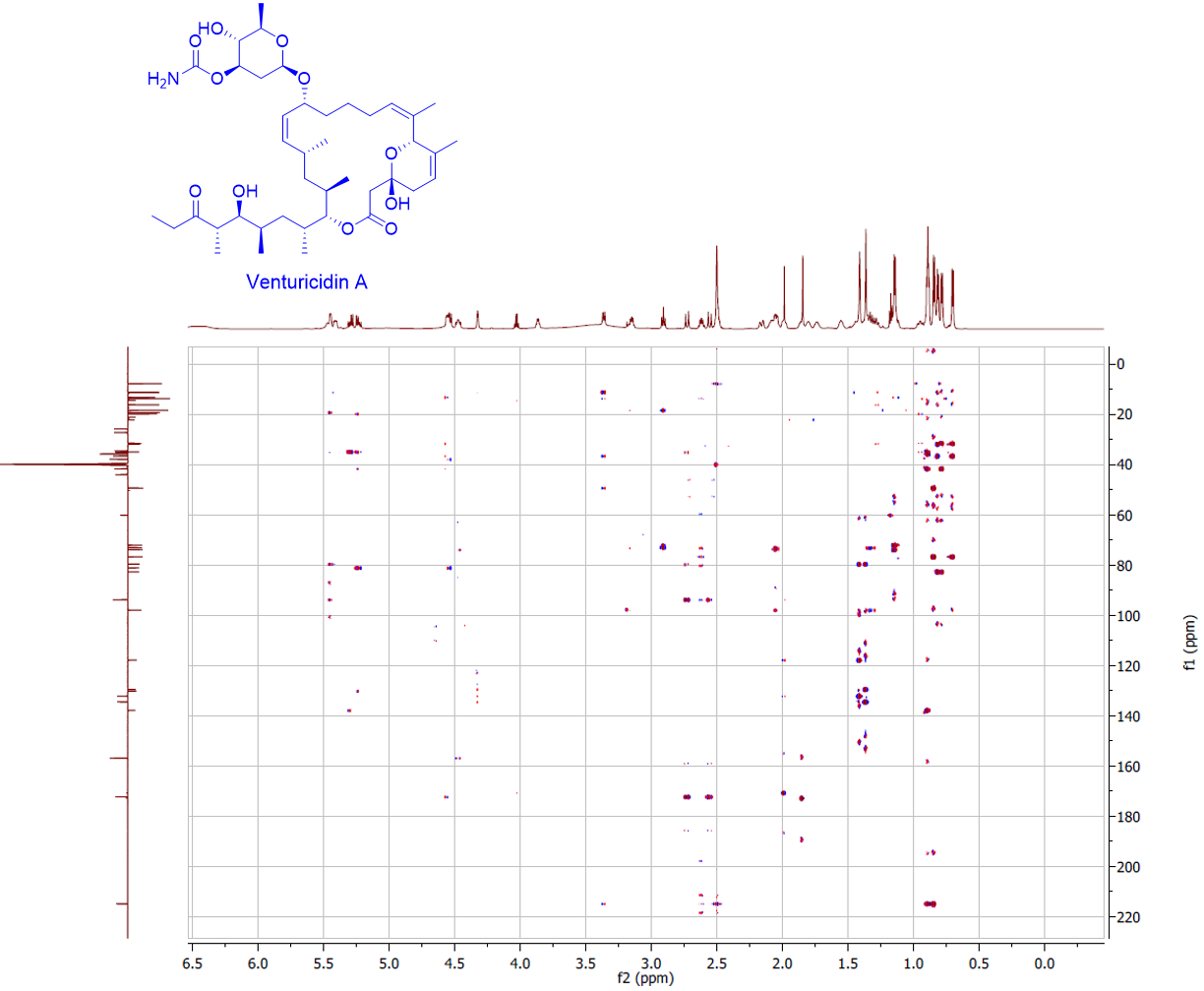
**

**Figure S8.** High resolution mass spectra of venturicidin A


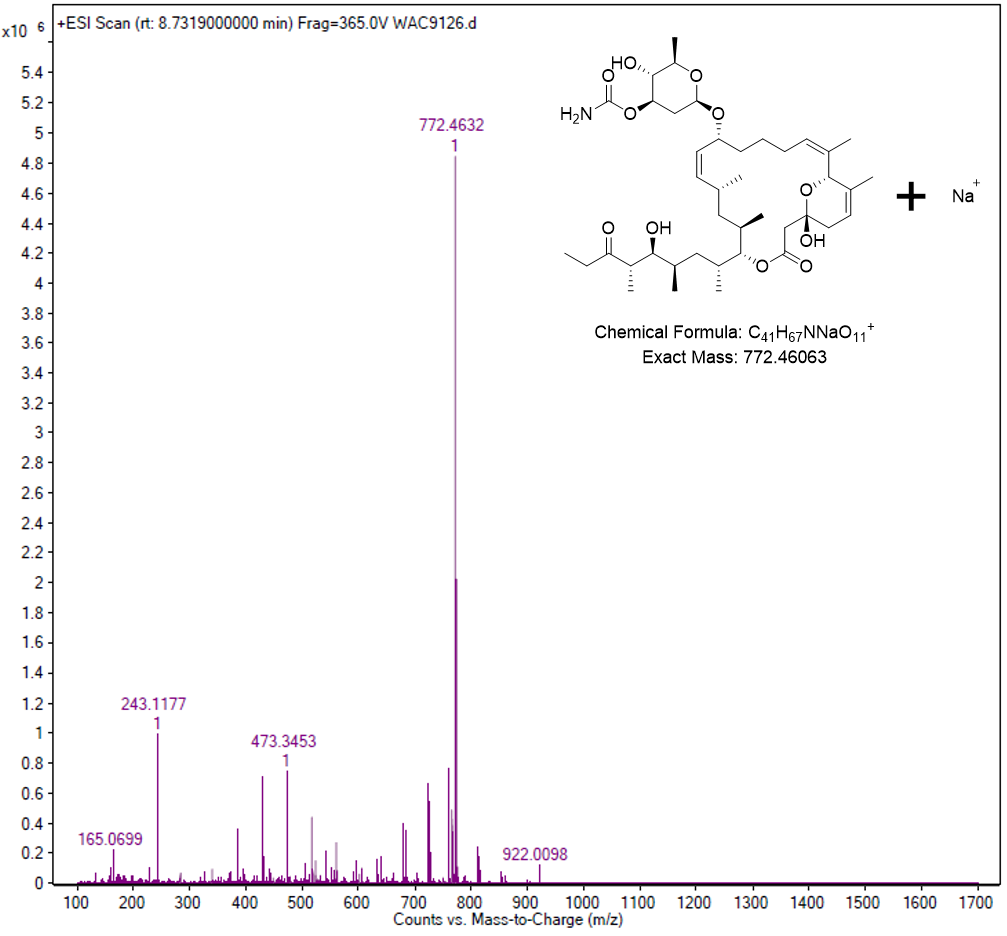


**Figure S9.** High resolution mass spectra of venturicidin C

**
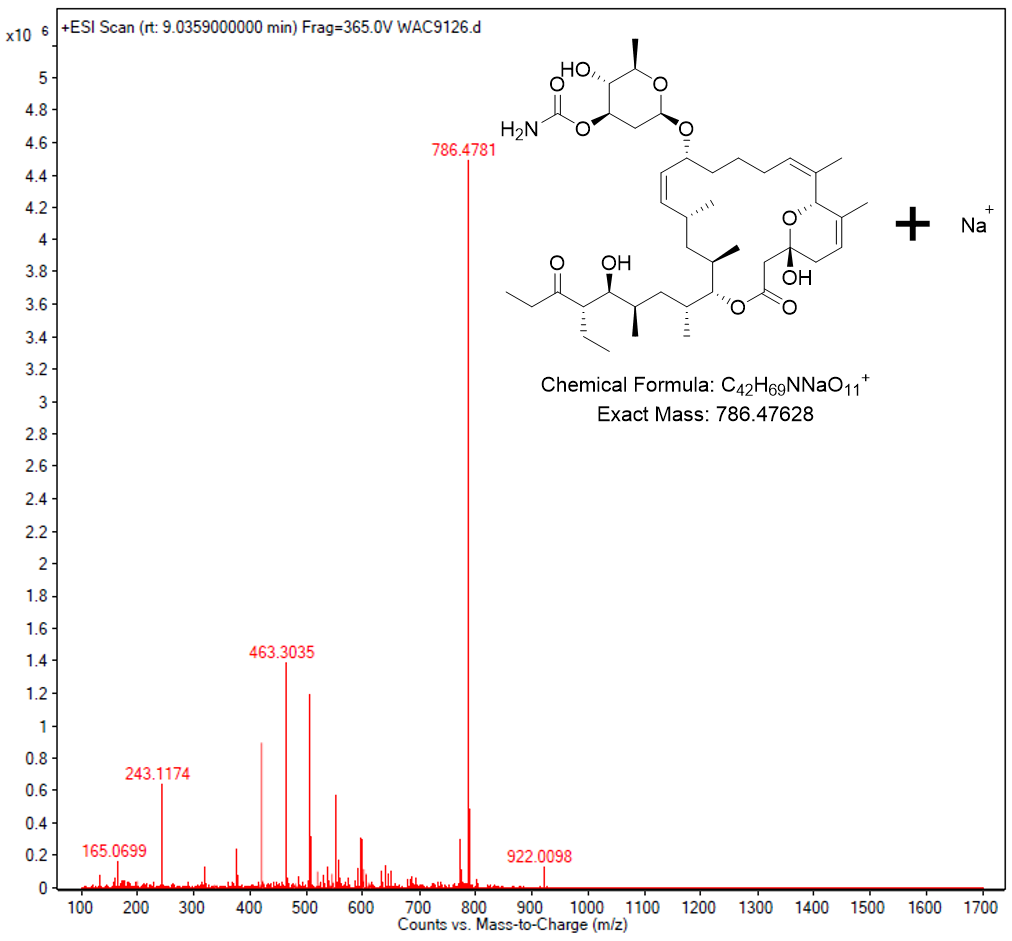
**

**Figure S10.** Growth inhibitory action of venturicidin A against CMRSA-1 (A) and CMRSA-3 (B)


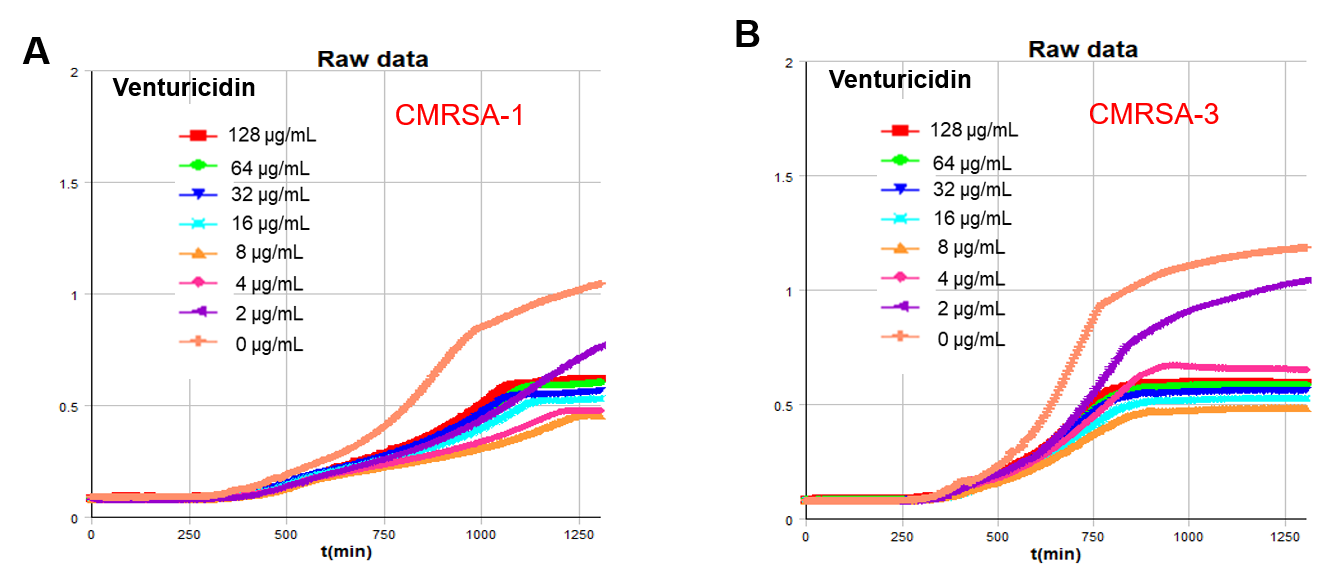


**Figure S11.** Combination studies with other classes of antibiotics against MRSA C1014 and *A. baumannii* C0412. Venturicidin A potentiates polymyxin B against MRSA, which is generally intrinsically resistant to polymyxins. Dark regions represent higher cell density.


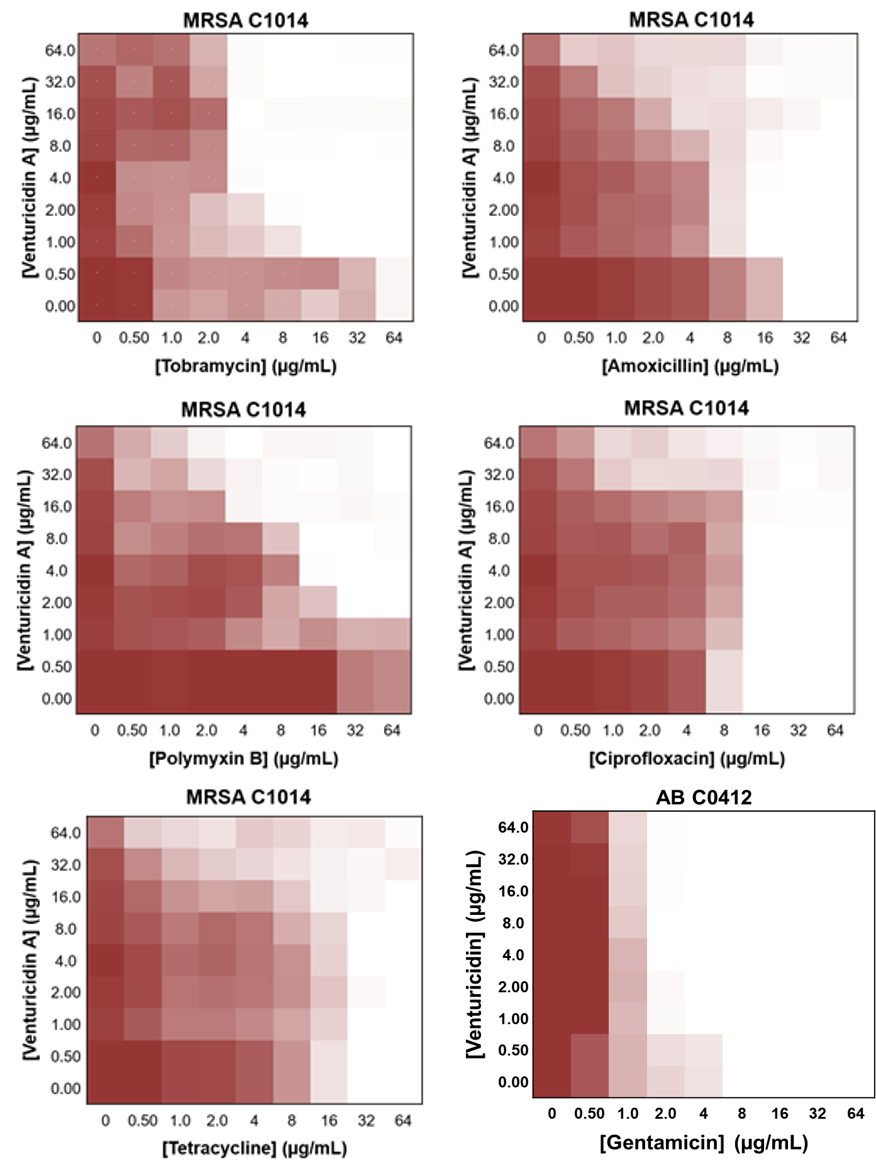


**Figure S12.** Interference of CCCP with DiSC_3_(5) fluorescence. (A) Fluorescence intensity of 1 μM DiSC_3_(5) in buffer containing 250 mM sucrose, 5 mM MgSO_4_, and 10 mM potassium phosphate (pH 7.0) upon addition of protonophore CCCP (2 µg/mL and 6 µg/mL). The time point of addition is highlighted with an arrow. (B) Fold of reduction in fluorescence intensity (VentA at 16 µg/mL and CCCP at 2 µg/mL) in absence and presence of the bacterial cells.


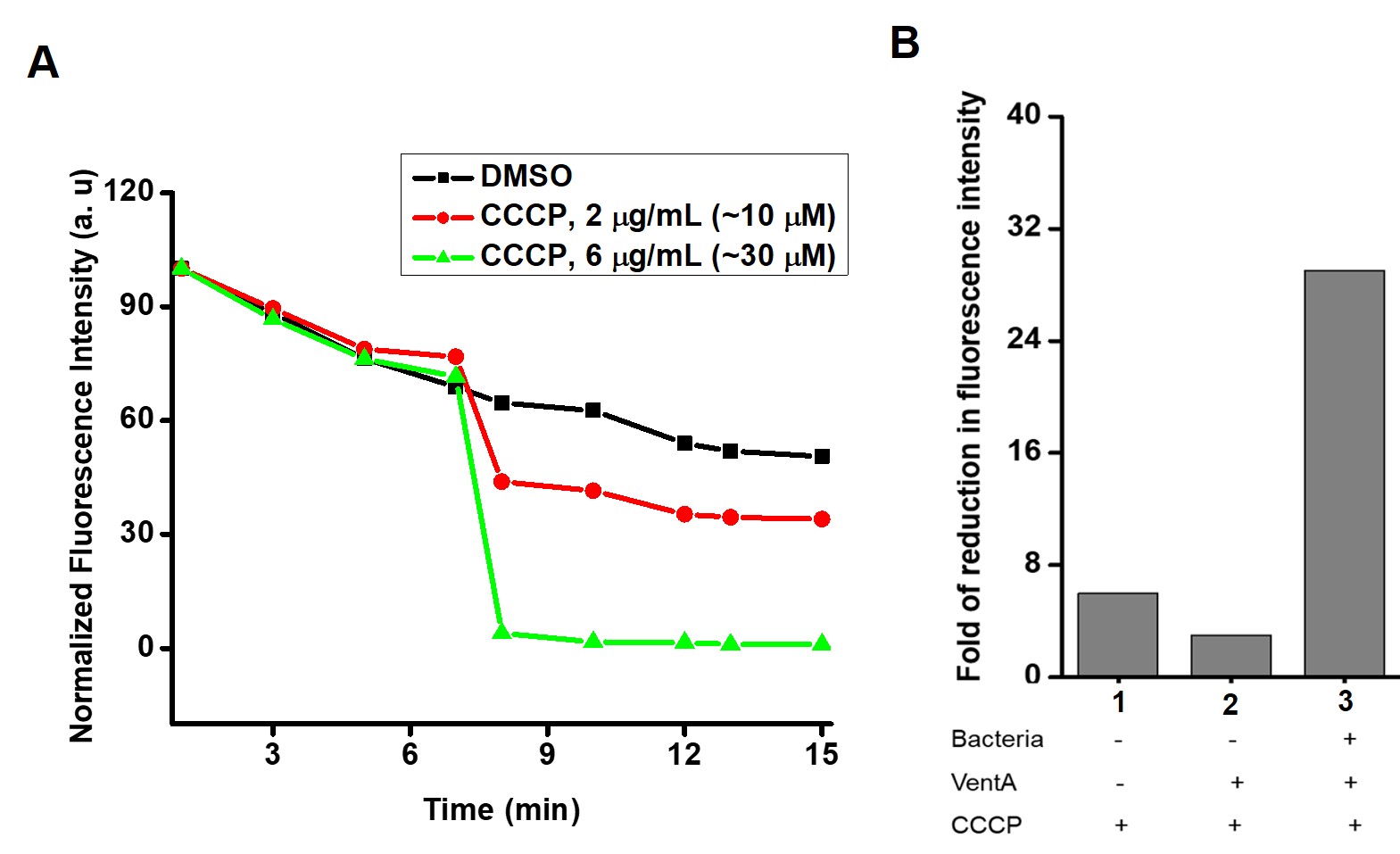


**Figure S13.** HPLC chromatogram of dansylation of standard gentamicin.

**
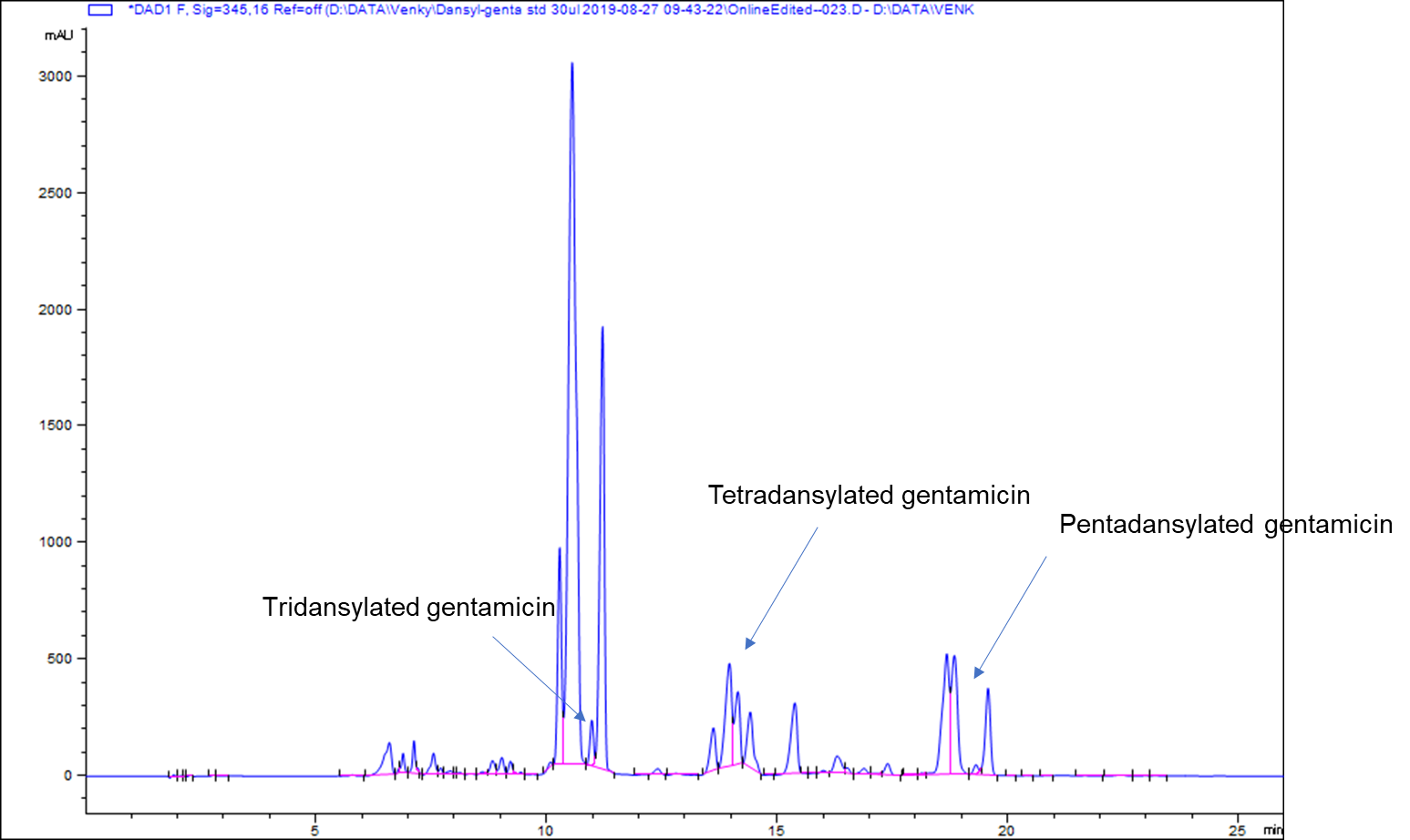
**

**Figure S14.** HPLC chromatogram of tetra- and penta-dansyl gentamicin.

**
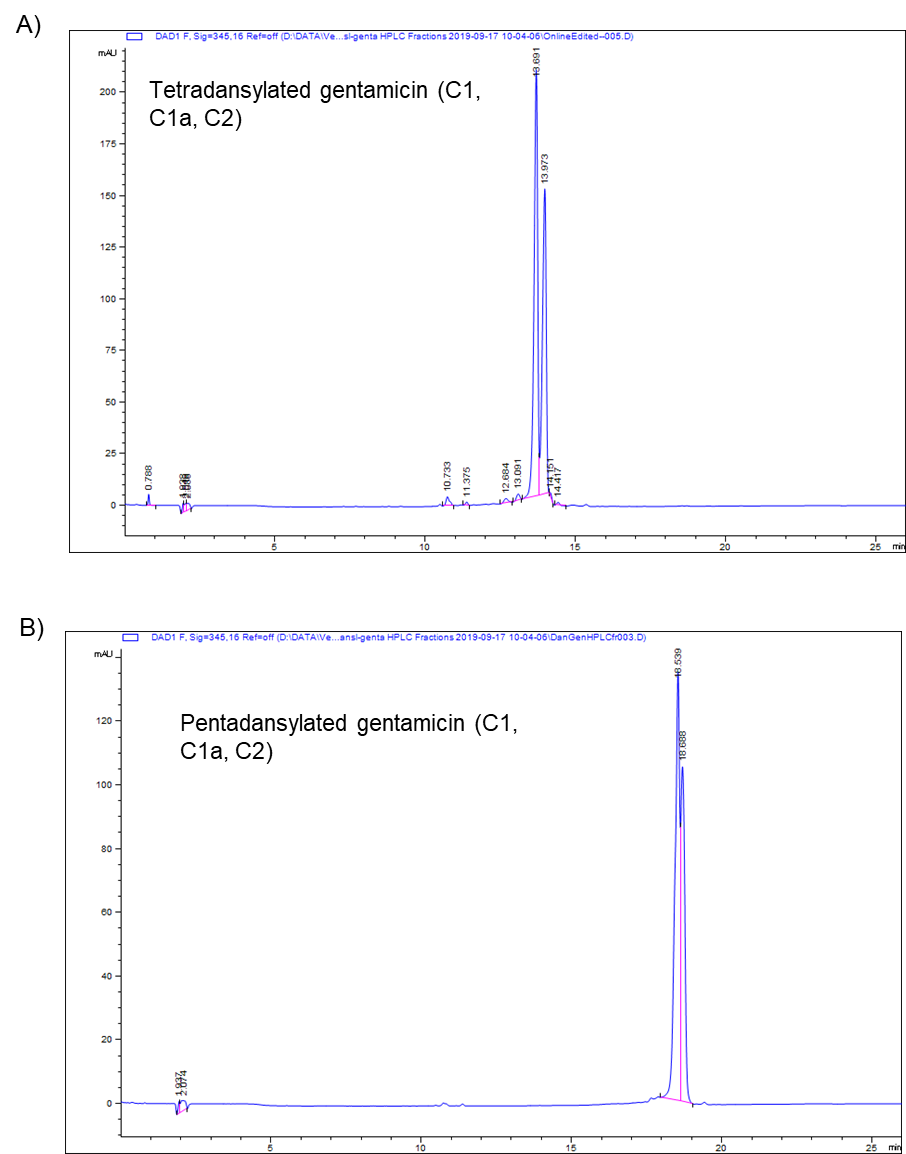
**

**Figure S15.** High-resolution mass spectral data of tetra- and penta-dansyl gentamicins.


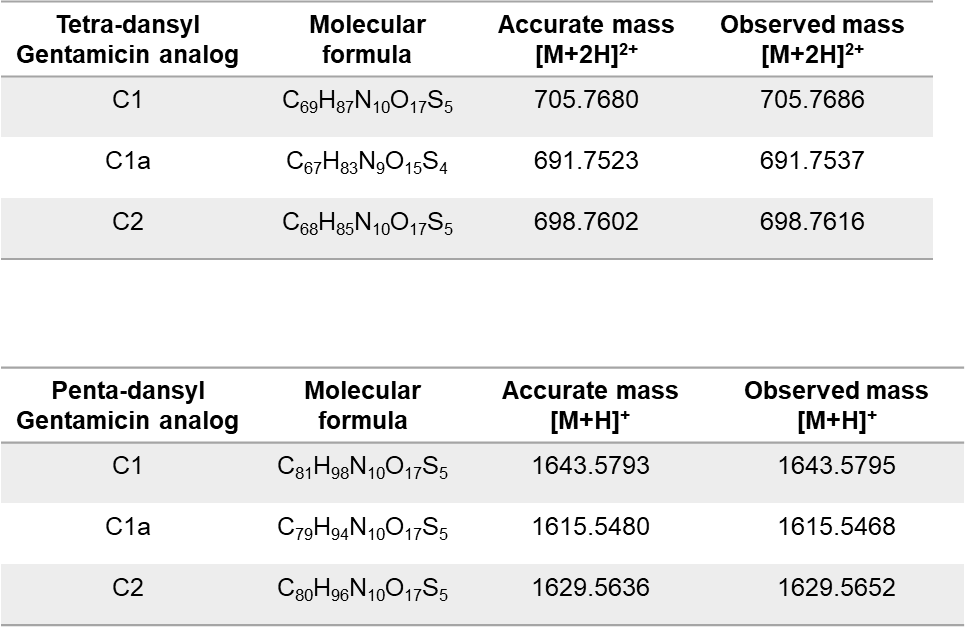


**Figure S16.** Intracellular accumulation of venturicidin A in presence and in the absence of gentamicin in *A. baumannii* C0286. Differences are considered statistically significant with a probability of *P* < 0.05.


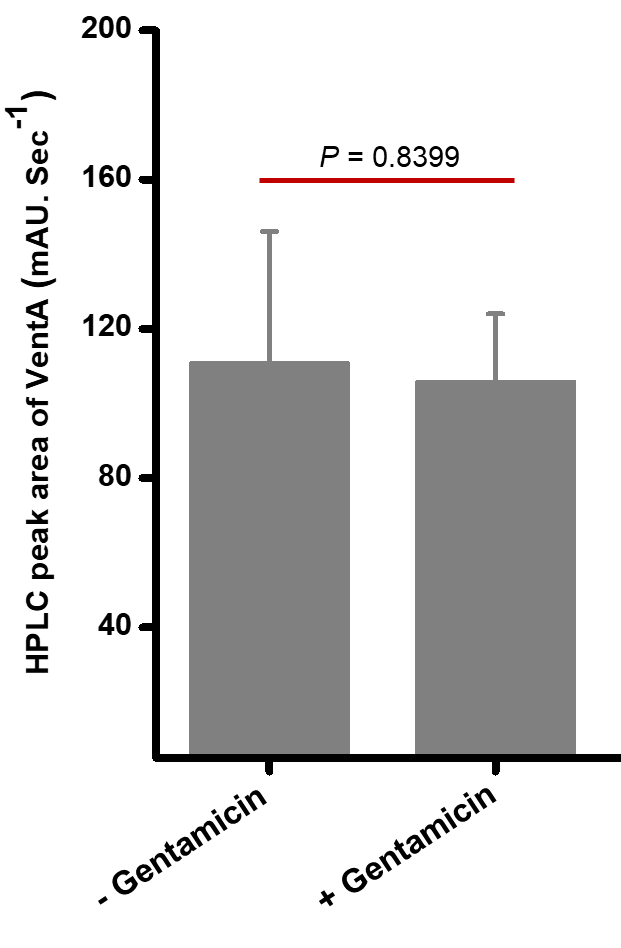


II. **Supplementary Tables**

**Table S1.** ^1^H and ^13^C NMR Data of venturicidin A

| No. | *δ*_H_ | *δ*_H_^a^ | *δ*_C_ | *δ*_C_^a^ |
| --- | --- | --- | --- | --- |
| 1 |  |  | 172.3, C | 171.9 |
| 2 | 2.71 – 2.74 (d) | 2.90 – 2.40 (m) | 44.2, CH_2_ | 43.6 |
| 3 | - | - | 93.9, C | 93.4 |
| 4 | 2.16 (m), 1.44 (m) | 2.08 (m), 1.50 (m) | 35.1, CH_2_ | 34.6 |
| 5 | 5.46 (brm) | 5.48 (brm) | 117.8, CH | 117.5 |
| 6 | - | - | 132.2, C | 131.8 |
| 6-CH_3_ | 1.41 (s) | 1.41 (s) | 19.3, CH_3_ | 18.9 |
| 7 | 4.33 (s) | 4.32 (brs) | 79.4, CH | 79.2 |
| 8 | - | - | 134.5, C | 134.1 |
| 8-CH_3_ | 1.37 (s) | 1.36 (s) | 11.2, CH_3_ | 10.7 |
| 9 | 5.41 (m) | 5.41 (m) | 130.0 | 129.9 |
| 10 | 2.06 (m), 1.86 (m) | 2.10 (m), 1.70 (m) | 27.3, CH_2_ | 26.9 |
| 11 | 1.39 (m), 1.16 (m) | 1.50 – 1.20 (m) | 25.8, CH_2_ | 25.5 |
| 12 | 2.09 (m) | 2.10 (m) | 34.8, CH_2_ | 34.3 |
| 13 | 3.87 (m) | 3.86 (m) | 81.2, CH | 82.3 |
| 14 | 5.29 (dd) | 5.31 (dd) | 130.3, CH | 129.1 |
| 15 | 5.26 (dd) | 5.22 (dd) | 137.8, CH | 137.4 |
| 16 | 2.09 (m) | 2.10 (m) | 35.0, CH | 34.8 |
| 16-CH_3_ | 0.90 (d) | 0.83 (d) | 19.8, CH_3_ | 19.4 |
| 17 | 1.14 (m), 0.95 (m) | 1.30 (m), 0.96 (m) | 41.7, CH_2_ | 41.3 |
| 18 | 1.55 (m) | 2.2 – 1.7 (m) | 31.7, CH | 31.2 |
| 18-CH_3_ | 0.84 (d) | 0.78 (d) | 13.7, CH_3_ | 12.8 |
| 19 | 4.56 (m) | 4.49 (m) | 82.7, CH | 80.8 |
| 20 | 1.74 (m) | 1.80 (m) | 31.9, CH | 31.5 |
| 20-CH_3_ | 0.82 (d) | 0.80 (d) | 16.3, CH_3_ | 15.8 |
| 21 | 1.33 (m), 0.84 (m) | 1.55 (m), 0.95 (m) | 37.9, CH_2_ | 36.2 |
| 22 | 1.80 (m) | 1.70 (m) | 31.5, CH | 31.1 |
| 22-CH_3_ | 0.71 (d) | 0.70 (d) | 11.3, CH_3_ | 10.9 |
| 23 | 3.38 (m) | 3.15 (m) | 76.7, CH | 76.3 |
| 23-OH | - | 5.03 (d) | - | - |
| 24 | 2.62 (m) | 3.15 (m) | 49.4, CH | 48.9 |
| 24-CH_3_ | 0.85 (d) | 0.88 (d) | 13.8, CH_3_ | 13.4 |
| 25 | - | - | 214.8, C | 214.5 |
| 26 | 2.51 (m) | 2.90 – 2.40 (m) | 35.8, CH_2_ | 35.4 |
| 27 | 0.9 (t) | 0.89 (t) | 7.9, CH_3_ | 7.4 |
| 1′ | 4.55 (brd) | 4.59 (brd) | 98.07, CH | 97.6 |
| 2′ | 2.06 (m), 1.32 (m) | 2.13 (m), 1.30 (m) | 37.9, CH_2_ | 37.5 |
| 3′ | 4.48 (m) | 4.43 (m) | 73.11, CH | 72.7 |
| 3′-CONH_2_ | 6.45 (brs) | 6.46 (brs) | 156.7, C | 156.5 |
| 4′ | 2.91 (m) | 3.36 (brm) | 73.8, CH | 73.5 |
| 5′ | 3.16 (m) | 3.36 (m) | 72.1, CH | 71.7 |
| 5′-CH_3_ | 1.15 (d) | 1.13 (d) | 18.4, CH_3_ | 18.0 |

*δ* is measured in p. p. m. NMR experiments were recorded in DMSO-D_6_. ^a^NMR data of venturicidin A from literature for comparison.^1^

**Table S2.** Antibacterial activity of purified venturicidin A against a range of bacterial pathogens

**
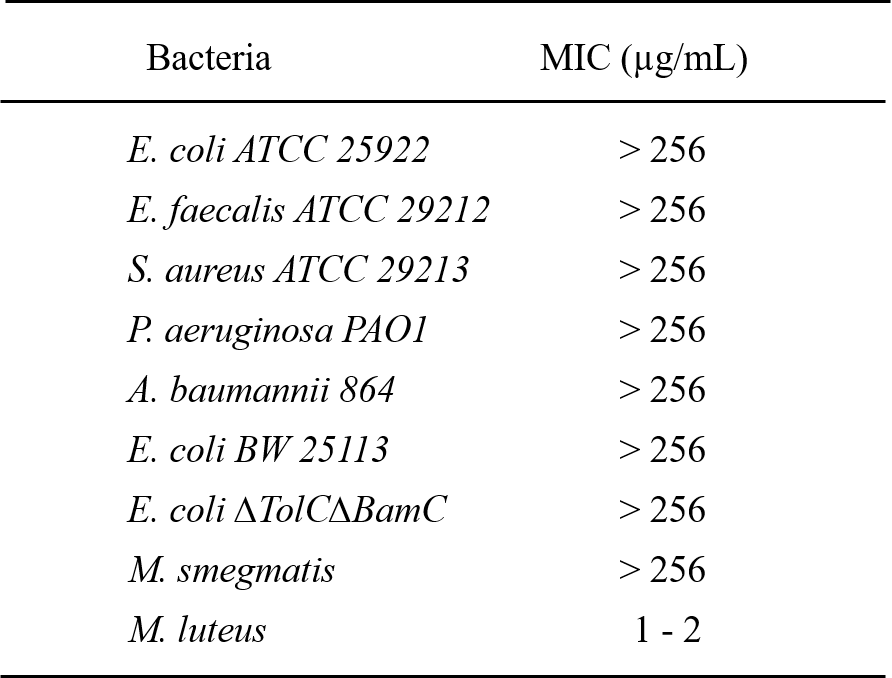
**

**Table S3.** Aminoglycoside resistance mechanisms of the clinical isolates tested


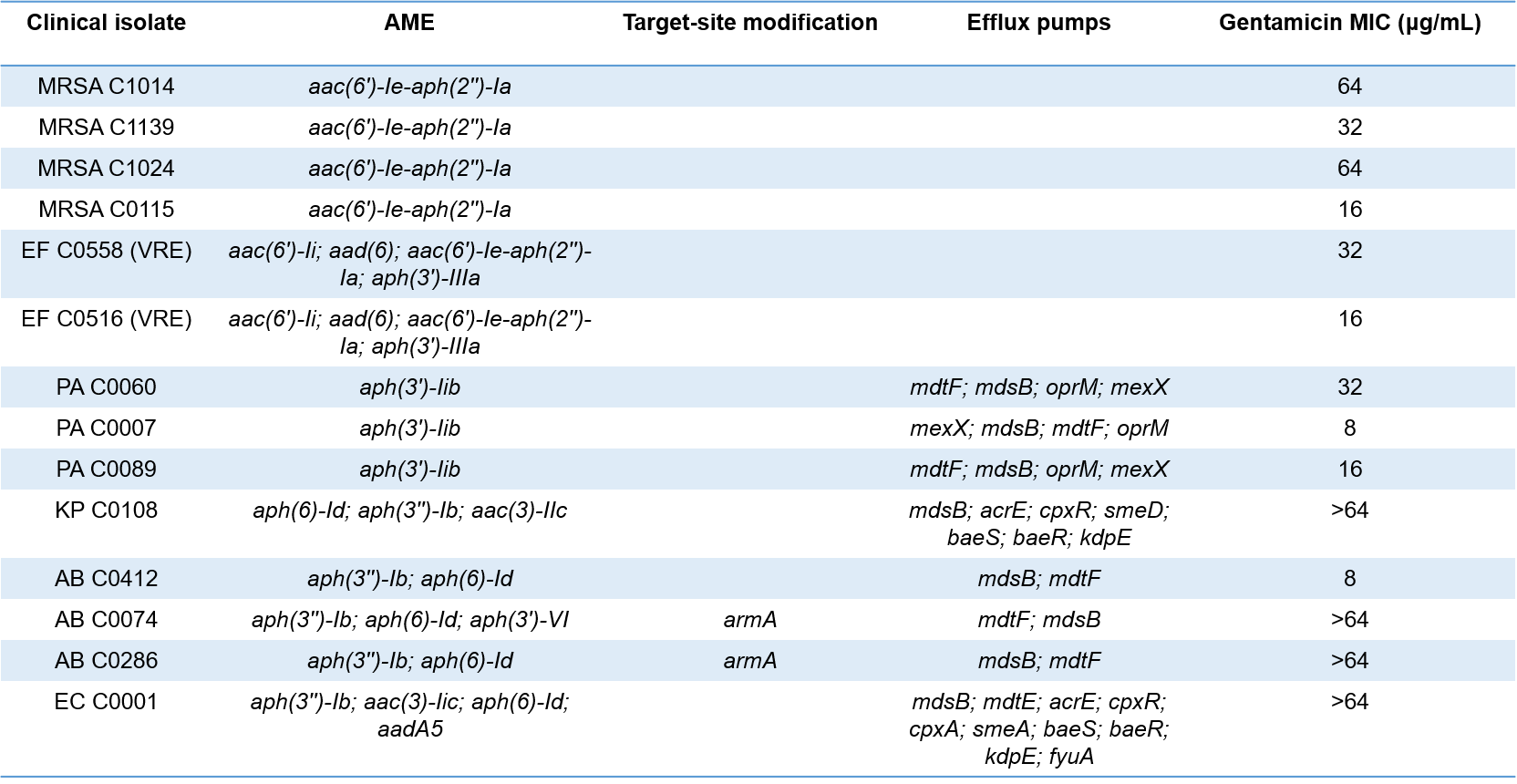


REFERENCES

1. Shaaban, K. A. *et al.* Venturicidin C, a new 20-membered macrolide produced by Streptomyces sp. TS-2-2. *J. Antibiot.* (Tokyo) **67**, 223-230 (2014).
